# Loss of membrane‐bound lytic transglycosylases increases outer membrane permeability and *β*‐lactam sensitivity in *Pseudomonas aeruginosa*

**DOI:** 10.1002/mbo3.286

**Published:** 2015-09-15

**Authors:** Ryan P. Lamers, Uyen T. Nguyen, Ylan Nguyen, Ryan N. C. Buensuceso, Lori L. Burrows

**Affiliations:** ^1^ Department of Biochemistry and Biomedical SciencesMichael G. DeGroote Institute for Infectious Disease ResearchMcMaster UniversityHamiltonOntarioCanada

**Keywords:** Antibiotic resistance, Braun's lipoprotein, cell envelope permeability, peptidoglycan‐associated lipoprotein, *β*‐lactam

## Abstract

The opportunistic pathogen *Pseudomonas aeruginosa* is a leading cause of nosocomial infections. Its relatively impermeable outer membrane (OM) limits antibiotic entry, and a chromosomally encoded AmpC *β*‐lactamase inactivates *β*‐lactam antibiotics. AmpC expression is linked to peptidoglycan (PG) recycling, and soluble (sLT) or membrane‐bound (mLT) lytic transglycosylases are responsible for generating the anhydromuropeptides that induce AmpC expression. Thus, inhibition of LT activity could reduce AmpC‐mediated *β*‐lactam resistance in *P. aeruginosa*. Here, we characterized single and combination LT mutants. Strains lacking SltB1 or MltB had increased *β*‐lactam minimum inhibitory concentrations (MICs) compared to wild type, while only loss of Slt decreased MICs. An *sltB1 mltB* double mutant had elevated *β*‐lactam MICs compared to either the *sltB1* or *mltB* single mutants (96 vs. 32 *μ*g/mL cefotaxime), without changes to AmpC levels. Time–kill assays with *β*‐lactams suggested that increased MIC correlated with a slower rate of autolysis in the *sltB1 mltB* mutant – an antisuicide phenotype. Strains lacking multiple mLTs were more sensitive to *β*‐lactams and up to 16‐fold more sensitive to vancomycin, normally incapable of crossing the OM. Multi‐mLT mutants were also sensitive to bile salts and osmotic stress, and were hyperbiofilm formers, all phenotypes consistent with cell envelope compromise. Complementation with genes encoding inactive forms of the enzymes – or alternatively, overexpression of Braun's lipoprotein – reversed the mutants' cell envelope damage phenotypes, suggesting that mLTs help to stabilize the OM. We conclude that *P. aeruginosa* mLTs contribute physically to cell envelope stability, and that Slt is the preferred target for future development of LT inhibitors that could synergize with *β*‐lactams.

## Introduction

*Pseudomonas aeruginosa* is a Gram‐negative opportunistic pathogen and frequent cause of hospital‐acquired infections. It belongs to the ESKAPE group of pathogens (with *Enterococcus faecium*,* Staphylococcus aureus*,* Klebsiella pneumoniae*,* Acinetobacter baumanii*, and *Enterobacter* spp.) for which treatment options are dwindling (Rice 2008). The discovery and development of novel antibiotics and antibiotic adjuvants is urgently required for treatment of these and other pathogens (Rice 2008; Boucher et al. 2009; Davies and Davies 2010). Among the mechanisms that contribute to antibiotic resistance in *P. aeruginosa* are the inducible expression of a chromosomally encoded AmpC *β*‐lactamase and reduced outer membrane (OM) permeability (Lister et al. 2009). The factors influencing OM characteristics (Cascales et al. 2002; Nikaido 2005; Ruiz et al. 2005) and AmpC induction (Jacobs et al. 1994; Kraft et al. 1999; Moya et al. 2009) remain incompletely understood.

In most Gram‐negative bacteria, the cell wall consists of a thin layer of peptidoglycan (PG) composed of alternating *N*‐acetylmuramic acid (NAM) and *N*‐ acetylglucosamine (NAG) glycan polymers, cross‐linked by stem peptides on the NAM residues (Johnson et al. 2013). The PG is located in the periplasm between the inner membrane (IM) and OM, and is connected to both via interactions with IM PG biosynthetic complexes and OM lipoproteins such as Braun's lipoprotein (Lpp) (Silhavy et al. 2010). PG maintenance and turnover are dynamic processes that rely on the coordinated efforts of multiple enzymes, including high‐molecular‐weight penicillin‐binding proteins (PBPs) that form the *β*(1,4)‐glycosidic bond between NAM and NAG, and cross‐link the stem peptides via their transglycosylase and transpeptidase activities, respectively. Low‐molecular‐weight PBPs have endopeptidase and/or carboxypeptidase activities that control the extent of crosslinking, while lytic transglycosylases (LTs) cleave the glycosidic bond between NAM and NAG with the concomitant formation of 1,6‐anhydromuropeptides (anhMPs), allowing for elongation and separation of daughter cells after cell division (Priyadarshini et al. 2006; Scheurwater et al. 2008). Under normal conditions, a proportion of anhMPs are shuttled back to the cytoplasm via the IM permease, AmpG, where they undergo processing by the *β*‐*N*‐acetylglucosaminidase, NagZ, and the amidase, AmpD, for recycling into nascent PG (Jacobs et al. 1994).

Changes in PG metabolism can impact antibiotic susceptibility in multiple ways. Inhibition of PBP transpeptidase function by *β*‐lactams leads to an imbalance between insertion of new material and cleavage of the existing cell wall by lytic enzymes, ultimately leading to loss of cellular integrity (Cho et al. 2014). In some Gram‐negative species, including *P. aeruginosa*, this autolytic activity leads to increased cytosolic accumulation of anhMPs that bind to the transcriptional activator, AmpR, inducing AmpC expression (Jacobs et al. 1994; Mark et al. 2011). Inactivation of AmpD – or its homologs AmpDh2 or AmpDh3 – increases cytosolic anhMP concentration and induces AmpC expression (Juan et al. 2006), while inactivation of AmpG prevents anhMPs from entering the cytoplasm, thereby preventing AmpC induction (Zhang et al. 2010; Zamorano et al. 2011). Inactivation of NagZ also prevents AmpC induction (Zamorano et al. 2010) by preventing processing of anhMPs to the form required for AmpR activation. Given the essential role of anhMPs in AmpC expression, inactivation of LTs has the potential to reduce the rate of anhMP production, thus preventing induction. However, LT inactivation could also delay autolysis, increasing the length of time that it takes for cells to accumulate sufficient damage to cause death and thus effectively increasing resistance. A clearer picture of the contributions of individual LTs to resistance would allow for more targeted drug development.

In *P. aeruginosa*, chemical or genetic inactivation of PBP4 – a D,D‐carboxypeptidase – also leads to increased *β*‐lactam resistance via induction of AmpC expression (Moya et al. 2009; Alvarez‐Ortega et al. 2010), presumably due to an increase in cytoplasmic levels of anhMPs with pentapeptide stems. Combining a PBP4 (*dacB*) mutation with loss of a specific LT – SltB1 – further increased resistance and AmpC expression via mechanisms that remain unclear, as *sltB1* mutants had wild‐type AmpC expression (Cavallari et al. 2013). Whether the loss of other LTs in a PBP4‐deficient background might similarly amplify *β*‐lactam resistance is unknown.

LTs are potential targets for novel antibiotics or antibiotic adjuvants (Bernal et al. 2013), but lack of systematic analyses of LT function, difficulties associated with studying enzymes that act on an insoluble substrate, and the apparent redundancy of these enzymes in many bacteria has hampered inhibitor development (Lee et al. 2013). LTs were classified into four families based on sequence similarity and the presence of consensus motifs (Blackburn and Clarke 2001; Scheurwater et al. 2008). The physiological roles of individual LTs – or of LTs belonging to specific families – remain largely unknown. Loss of specific Family 1 LTs in *P. aeruginosa* increased *β*‐lactam sensitivity, while loss of specific Family 3 LTs decreased *β*‐lactam sensitivity (Cavallari et al. 2013). Whether membrane‐bound (mLTs) and soluble (sLTs) lytic transglycosylases in general have different roles in antibiotic resistance is not clear.

Here, we examined the contributions of individual LTs, LT families, and mLTs versus sLTs to *β*‐lactam sensitivity in *P. aeruginosa* by systematically deleting the genes encoding these enzymes. In most cases, the loss of one or few LTs caused modest changes in minimum inhibitory concentrations (MICs), while the loss of all mLTs caused extreme *β*‐lactam and vancomycin sensitivity. Slt was the only enzyme whose individual loss decreased *β*‐lactam MICs (Cavallari et al. 2013; Cho et al. 2014). Increased *β*‐lactam MICs due to loss of Family 3 LTs – all but one of which are sLTs – were associated with slower rates of death. This study suggests that modest changes in MICs caused by loss of single enzymes contribute to stepwise changes in overall resistance (Juan et al. 2006; Alvarez‐Ortega et al. 2010; Breidenstein et al. 2011; Fernandez et al. 2011) and reveals the potential of Slt as a target for combination therapies with *β*‐lactam antibiotics.

## Materials and Methods

### Bacterial strains, plasmids, and mutants

The nine *P. aeruginosa* LT genes previously identified by bioinformatic methods (Blackburn and Clarke 2001; Legaree and Clarke 2008) were analyzed in this study. A 10th LT, RlpA (rare lipoprotein A) was identified by Jorgenson et al. (2014) and despite having only weak structural similarity to MltA from *Escherichia coli* and no activity on wild‐type PG, was shown to preferentially degrade PG devoid of peptide stems, contributing to daughter cell separation. Under normal growth conditions, the mutant was reported to have a wild‐type phenotype. Bacterial strains and plasmids used in this study are listed in Table 1. *Pseudomonas aeruginosa* mutant strains were created using either a Flp‐FRT gene disruption system or unmarked gene deletion (Hoang et al. 1998). Genes were disrupted by FRT insertion following previously described methods (Cavallari et al. 2013). To create unmarked gene deletions, appropriate pEX18Gm constructs were transferred to *P. aeruginosa* strains of interest by conjugation with donor strain *E. coli* SM10, and mating mixtures were plated on *Pseudomonas* isolation agar containing 100 *μ*g/mL gentamicin (Gm) to counter‐select the donor. Gm‐resistant colonies were plated on modified Luria–Bertani agar (LBA), no salt, 5% sucrose to select for double recombinants that lost the *sacB*‐expressing pEX18Gm suicide vector. Gm‐sensitive mutants with insertion or deletion in the gene of interest were confirmed by PCR using gene‐specific and flanking primers (see Table S1).

**Table 1:** Bacterial strains and plasmids used in this study

Strain or plasmid	Description	Reference or source	Notes
Strains			
PAO1	*Pseudomonas aeruginosa* wild‐type strain	Li et al. (1998), Lamers et al. (2013)	
PAO1 *mltA*	WT with *mltA* deletion (PA1222)	This study	Family 2
PAO1 *mltB*	WT with *mltB* deletion (PA4444)	This study	Family 3
PAO1 *mltD*	WT with *mltD* deletion (PA1812)	This study	Family 1D
PAO1 *mltF*	WT with *mltF* deletion (PA3764)	This study	Family 1E
PAO1 *mltF2*	WT with *mltF2* deletion (PA2865)	This study	Family 1E
PAO1 *slt*	WT with *slt* deletion (PA3020)	This study	Family 1A
PAO1 *sltB1*	WT with *FRT* scar at nucleotide 577 of *sltB1* (PA4001)	Cavallari et al. (2013)	Family 3
PAO1 *sltG*	WT with *sltG* deletion (PA1171)	This study	Family 3
PAO1 *sltH*	WT with *sltH* deletion (PA3992)	This study	Family 3
PAO1 *sltB1*/*slt*	*sltB1* mutant with *slt* deletion	This study	
PAO1 *sltB1*/*G*	*sltB1* mutant with *sltG* deletion	This study	
PAO1 *sltB1*/*H*	*sltB1* mutant with *sltH* deletion	This study	
PAO1 *sltB1*/*mltB*	*sltB1* mutant with *slt* deletion	This study	
PAO1 *sltB1*/*G*/*mltB*	*sltB1*/*mltB* mutant with *sltG* deletion	This study	
PAO1 *sltB1*/*G*/*H*	*sltB1*/*H* mutant with *sltG* deletion	This study	
PAO1 *sltB1*/*G*/*H*/*slt*	*sltB1*/*G*/*H* mutant with *slt* deletion	This study	Strain lacking all soluble LTs
PAO1 *sltB1*/*G*/*H*/*mltB*	*sltB1*/*G*/*H* mutant with *mltB* deletion	This study	Strain lacking all Family 3 LTs
PAO1 *slt*/*mltF*	*slt* mutant with *mltF* deletion	This study	
PAO1 *slt*/*mltB*	*slt* mutant with *mltB* deletion	This study	
PAO1 *mltB*/*F*	*mltB* mutant with *mltF* deletion	This study	
PAO1 *mltD*/*F*	*mltD* mutant with *mltF* deletion	This study	
PAO1 *mltB*/*D*/*F*	*mltD*/*F* mutant with *mltB* deletion	This study	
PAO1 *mltB*/*F*/*F2*	*mltB*/*F* mutant with *mltF2* deletion	This study	
PAO1 *mltD*/*F*/*F2*	*mltD*/*F* mutant with *mltF2* deletion	This study	
PAO1 *mltA*/*B*/*F*	*mltB*/*F* mutant with *mltA* deletion	This study	
PAO1 *mltB*/*D*/*F*/*F2*	*mltD*/*F*/*F2* deletion with *mltB* deletion	This study	
PAO1 *mltD*/*F*/*F2*/*slt*	*mltD*/*F*/*F2* deletion with *slt* deletion	This study	Strain lacking all Family 1 LTs
PAO1 mltA/*B*/*D*/*F*/*F2*	*mltB*/*D*/*F*/*F2* deletion with *mltA* deletion	This study	Strain lacking all membrane‐bound LTs
PAO1 *ampC*	WT with *FRT* scar at nucleotide 1070 of *ampC* (PA4110)	Cavallari et al. (2013)	
PAO1 *sltB1*/*ampC*	*sltB1* mutant with *FRT* scar at nucleotide 1070 of *ampC*	Cavallari et al. (2013)	
PAO1 *sltH*/*ampC*	*sltH* mutant with *FRT* scar at nucleotide 1070 of *ampC*	This study	
PAO1 *mltB*/*ampC*	*mltB* mutant with *FRT* scar at nucleotide 1070 of *ampC*	This study	
PAO1 *mltD*/*ampC*	*mltD* mutant with *FRT* scar at nucleotide 1070 of *ampC*	This study	
PAO1 *mltF2*/*ampC*	*mltF2* mutant with *FRT* scar at nucleotide 1070 of *ampC*	This study	
PAO1 *dacB*	WT with *FRT* scar at nucleotide 168 of *dacB* (PA3047)	Lamers et al. (2013)	
PAO1 *dacB*/*slt*	*dacB* mutant with *slt* deletion	This study	
PAO1 *sltB1*/*dacB*	*sltB1* mutant with FRT scar at nucleotide 168 of *dacB*	Cavallari et al. (2013)	
PAO1 *sltB1*/*G*/*H*/*mltB*/*dacB*	*sltB1*/*G*/*H*/*mltB* mutant with FRT scar at nucleotide 168 of *dacB*	This study	
PAO1 *sltG*/*H*/*mltB*/*dacB*	*sltB1*/*G*/*H*/*mltB*/*dacB* mutant with replacement of *sltB1*::FRT with wild‐type *sltB1*	This study	
PAO1 *sltB1*/*G*/*H*/*slt*/*dacB*	*sltB1*/*G*/*H*/*slt* mutant with FRT scar at nucleotide 168 of *dacB*	This study	
PAO1 *sltG*/*H*/*slt*/*dacB*	*sltB1*/*G*/*H*/*slt*/*dacB* mutant with replacement of *sltB1*::FRT with wild‐type *sltB1*	This study	
Plasmids			
pPS856	Source of *FRT‐*flanked gentamicin cassette; Gm^r^	Hoang et al. (1998)	
pEX18Ap	Suicide vector used for gene disruption; Ap^r^	Hoang et al. (1998)	
pEX18Gm	Suicide vector used for gene deletion; Gm^r^	Hoang et al. (1998)	
pFLP2	Suicide vector encoding Flp recombinase; Ap^r^	Hoang et al. (1998)	
pBADGr	pMLBAD backbone with *dhfr* replaced with *aacC1*; arabinose inducible; Gm^r^	Asikyan et al. (2008), Cavallari et al. 2013)	
pEX18Ap‐*sltB1*::FRTGmFRT	Suicide vector containing *sltB1* disrupted at nucleotide position 577 with *FRT‐*flanked gentamicin resistance cassette; Ap^r^ Gm^r^	Cavallari et al. (2013)	
pEX18Ap‐*ampC*::FRTGmFRT	Suicide vector containing *ampC* disrupted at nucleotide position 1070 with *FRT‐*flanked gentamicin resistance cassette; Ap^r^ Gm^r^	Cavallari et al. (2013)	
pEX18Ap‐*dacB*::FRTGmFRT	Suicide vector containing *dacB* disrupted at nucleotide position 168 with *FRT‐*flanked gentamicin resistance cassette; Ap^r^ Gm^r^	Lamers et al. (2013)	
pEX18Gm‐*mltA*	Suicide vector containing 500 nucleotides flanking each side of *mltA* for recombination; Gm^r^	This study	
pEX18Gm‐*mltB*	Suicide vector containing 500 nucleotides flanking each side of *mltB* for recombination; Gm^r^	This study	
pEX18Gm‐*mltD*	Suicide vector containing 500 nucleotides flanking each side of *mltD* for recombination; Gm^r^	This study	
pEX18Gm‐*mltF*	Suicide vector containing 500 nucleotides flanking each side of *mltF* for recombination; Gm^r^	This study	
pEX18Gm‐*mltF2*	Suicide vector containing 500 nucleotides flanking each side of *mltF2* for recombination; Gm^r^	This study	
pEX18Gm‐*sltB1*_WT_	Suicide vector containing wild‐type *sltB1*; Gm^r^	This study	
pEX18Gm‐*slt*	Suicide vector containing 500 nucleotides flanking each side of *slt* for recombination; Gm^r^	This study	
pEX18Gm‐*sltG*	Suicide vector containing 500 nucleotides flanking each side of *sltG* for recombination; Gm^r^	This study	
pEX18Gm‐*sltH*	Suicide vector containing 500 nucleotides flanking each side of *sltH* for recombination; Gm^r^	This study	
pBADGr‐OprI	pBADGr derivative containing OprI (Braun's lipoprotein, Lpp; PA2853) on an EcoRI to HindIII fragment; Gm^r^	This study	
pBADGr‐OprL	pBADGr derivative containing OprL (peptidoglycan‐associated lipoprotein, Pal; PA0973) on an EcoRI to HindIII fragment; Gm^r^	This study	

### Antibiotic sensitivity assays

Antibiotic sensitivity assays were performed using Etest strips (BioMérieux Canada, Inc., St. Laurent, Quebec, Canada) as previously described (Cavallari et al. 2013), and broth microdilution. For Etest assays, overnight bacterial cultures were subcultured 1:50 in Mueller–Hinton Broth (MHB; Becton, Dickinson and Company, Mississauga, Ontario, Canada) and grown to logarithmic phase at 37°C, with shaking at 200 rpm. Cultures were standardized to an optical density at 600 nm (OD_600 nm_) of 0.25 in MHB, and 100 *μ*L was evenly spread on Mueller–Hinton agar (MHA) and allowed to dry. Etest strips were overlaid and plates were incubated for 18 h at 37°C. As per the manufacturer's recommendations, MICs were determined to be the concentration at which the zone of inhibition intersected the Etest strip. Etest assays were performed three times independently and the modal MIC value was listed in Table 2. Variations in MICs between replicates were never more than 1.5‐fold; and thus, only mutants with MIC differences twofold or greater than wild type were tested further for reproducibility using the broth microdilution method.

**Table 2:** MICs for LT mutants of *β*‐lactam, fluoroquinolone, and glycopeptide antibiotics

Strain	Minimum inhibitory concentration (*μ*g/mL)^a^^,^ b						
	PP	CT	TZ	IP	CI	VA	PMB
PAO1	6 (4)	12 (8)	1 (2)	1	0.19	>256 (2048)	(1)
*ampC*	2 (1)	4 (4)	– (0.5)	0.5	nd	nd	nd
*dacB*	64 (32)	>256 (128)	16 (16)	–	nd	nd	nd
*dacB*/*slt*	32 (8)	128 (64)	8 (8)	–	nd	nd	nd
*slt*	3 (1)	6 (4)	0.5 (1)	–	–	–	nd
*slt* (E503A)	4 (1)	6 (4)	0.5 (1)	–	nd	nd	nd
*sltB1*	16 (8)	24 (16)	– (–)	–	–	–	nd
*sltB1*/*ampC*	4 (2)	4 (4)	– (1)	0.5	nd	nd	nd
*sltH*	12 (8)	32 (16)	– (–)	1.5	–	–	nd
*sltH*/*ampC*	4 (2)	16 (4)	– (1)	0.25	nd	nd	nd
*sltB1*/*mltB*	32 (16)	96 (32)	1.5 (–)	0.75	–	–	nd
*mltB*	12 (–)	32 (16)	1.5 (–)	1.5	–	–	nd
*mltB* (E162A)	8 (–)	16 (–)	– (–)	–	nd	nd	nd
*mltB*/*ampC*	8 (2)	16 (4)	– (–)	0.5	nd	nd	nd
*mltD*	12 (–)	24 (16)	– (–)	–	–	– (–)	(–)
*mltD* (E144A)	– (–)	16 (–)	– (–)	–	nd	nd	nd
*mltD*/*ampC*	– (2)	16 (4)	– (–)	0.5	nd	nd	nd
*mltF2*	12 (–)	32 (16)	– (–)	–	–	–	nd
*mltF2*/*ampC*	– (–)	8 (4)	–	0.5	nd	nd	nd
*mltB*/*D*/*F*	4 (2)	6 (4)	0.75 (1)	–	–	–	nd
*mltB*/*F*/*F2*	4 (2)	6 (4)	0.75 (1)	–	–	–	nd
*mltD*/*F*/*F2*	3 (1)	6 (4)	– (1)	0.75	–	– (512)	(–)
*mltB*/*D*/*F*/*F2*	3 (1)	4 (4)	– (1)	–	–	– (256)	(0.5)
*mltD*/*F*/*F2*/*slt*	1.5 (0.5)	2 (2)	0.5 (0.5)	0.5	0.125	128 (256)	(0.5)
*mltA*/*B*/*D*/*F*/*F2*	2 (0.5)	4 (4)	0.75 (0.5)	0.5	0.125	128 (128)	(0.5)

PP, piperacillin; CT, cefotaxime; TZ, ceftazidime; IP, imipenem; CI, ciprofloxacin; VA, vancomycin; PMB, Polymyxin B; nd, not done;–, MIC is same as wild type.

a MICs in blue are ≥twofold higher than wild type, while those in red are ≥twofold lower than wild type, , as confirmed by Etest and broth microdilution methods.

b MIC values in parentheses were determined using broth microdilution, while all other MIC values were determined by Etest.

Strains with Etest MIC differences twofold or greater than control – and their respective control strains – were tested further using broth microdilution following Clinical and Laboratory Standards Institute (CLSI) guidelines. For these assays, antibiotics were serially diluted twofold and 1 *μ*L of the desired concentration was added to the appropriate wells of a 96‐well microtitre plate. Overnight bacterial cultures were subcultured 1:50 in MHB and grown to logarithmic phase at 37°C, with shaking at 200 rpm. Cultures were standardized to ~4.0 × 10^5^ colony forming units per mL (CFU/mL) in MHB, and a 99 *μ*L inoculum was combined with the desired antibiotic in the appropriate wells of a 96‐well microtitre plate. Plates were covered with Mylar seals (Thermo Scientific, Mississauga, Ontario, Canada) and incubated at 37°C. MICs were determined as the concentration at which no visible growth was observed after 18 h incubation. Broth microdilution MIC assays were performed three times independently with three technical replicates each. Only reproducible MIC changes (i.e., confirmed by Etest and broth microdilution methods both) were listed as different than wild type in Table 2. We note that MIC differences of twofold are generally not regarded as clinically significant; however, modest changes in MIC may contribute to stepwise changes to resistance (Juan et al. 2006; Alvarez‐Ortega et al. 2010; Kumari et al. 2014), and therefore, we listed all reproducible MIC changes of twofold or greater in Table 2.

### AmpC *β*‐lactamase western blot analyses

Overnight bacterial cultures were subcultured 1:20 in 5 mL LB and grown to an OD_600 nm_ = 0.6 at 37°C and 200 rpm. For assays using chemical induction of AmpC, cultures were split 1:1 in 5 mL LB with or without 50 *μ*g/mL cefoxitin (inducer) and incubated for an additional 2 h at 37°C and 200 rpm. Cell cultures were then standardized to an OD_600 nm_ = 0.6 and 1 mL was centrifuged at 16,100*g* for 1 min. For assays using genetic induction of AmpC (i.e., via disruption of *dacB*), logarithmic‐phase cell cultures were standardized to an OD_600 nm_ = 0.6 and 1 mL was centrifuged at 16,100*g* for 1 min. Cell pellets were resuspended in 100 mL of sodium dodecyl sulfate (SDS) sample buffer (0.3 mol/L Tris‐HCl, pH 6.8, SDS [6.7% w/v], glycerol [10% v/v], 2‐mercaptoethanol [5.3% v/v] and bromophenol blue [0.2% w/v]), boiled for 10 min and stored at −20°C until immunoblotting.

For polyclonal antibody production, *P. aeruginosa ampC* (PA4110) lacking the first 78 nucleotides (amino acids 1–26) was cloned into pET151/D‐TOPO vector (Life Technologies, Mississauga, Ontario, Canada) and expressed following the manufacturer's instructions in *E. coli* BL21 cells at 37°C without induction. Cells were lysed by sonication and AmpC_Δ26_ was purified by nickel affinity chromatography, followed by Tobacco Etch Virus (TEV) protease cleavage and a second nickel affinity purification step to isolate untagged AmpC_Δ26_. Purified AmpC_Δ26_ was dialyzed (using Slide‐A‐Lyzer Dialysis Cassette, 10 kDa molecular weight cutoff; Thermo Scientific, Mississauga, Ontario, Canada) with phosphate‐buffered saline (PBS; pH 7.0), the protein concentration was adjusted to 1 mg/mL, and sample was submitted to Cedarlane Laboratories (Burlington, Ontario, Canada) for rabbit immunization.

Immunoblotting was performed by first separating samples on 12.5% SDS‐PAGE (polyacrylamide gel electrophoresis) gels at 150 V for 90 min followed by transfer to nitrocellulose membranes at 225 mA for 60 min. Membranes were blocked using 5% skim milk in PBS at 37°C for 60 min, followed by washing with PBS and incubation with *α*‐AmpC primary antibody (1/2000 dilution in PBS) overnight at 4°C. Membranes were washed with PBS and incubated with *α*‐rabbit secondary antibody conjugated to alkaline phosphatase (1/3000 dilution in PBS) for 1 h at 37°C followed by washing again with PBS. Membranes were developed in a solution containing 100 *μ*L nitro‐blue tetrazolium and 100 *μ*L 5‐bromo‐4‐chloro‐3‐indolyl phosphate (BCIP) in 10 mL of alkaline phosphatase buffer (100 mmol/L NaCl, 5 mmol/L MgCl_2_, 100 mmol/L Tris pH 9.5).

### Cell envelope integrity assays

Cell envelope integrity was tested by measuring bacterial survival on bile salts containing or high‐osmolarity media (Wang 2002; Korsak et al. 2005). Overnight bacterial cultures were subcultured 1:50 in LB and grown at 37°C and 200 rpm to an OD_600 nm_ = 0.6. For bile salt assays, cultures were serially diluted and 50 *μ*L spread on LBA, with or without 0.15% No. 3 bile salts (Sigma‐Aldrich, Oakville, ON, Canada). Plates were incubated overnight at 37°C. Percent survival was determined by enumerating CFUs from samples treated with bile salts compared to matched, untreated samples. For high‐osmolarity assays, logarithmic‐phase cultures were standardized to OD_600 nm_ = 0.1 in LB containing either 85.5 mmol/L or 2.5 mol/L NaCl and incubated for 2 h at room temperature. Cultures were then serially diluted followed by spreading 50 *μ*L on LBA and incubated overnight at 37°C. The percent survival of strains to high‐osmolarity conditions was determined by enumerating CFUs from samples treated with 2.5 mol/L NaCl compared to matched samples treated with 85.5 mmol/L NaCl. Cell envelope integrity assays were performed three times independently and statistical significance was assessed using a two‐tailed Student's *t*‐test. A *P* ≤ 0.05 was considered statistically significant.

### Biofilm assays

Biofilm assays were performed as previously described (Wenderska et al. 2011). Briefly, overnight cultures grown in PBS (137 mmol/L NaCl, 2.7 mmol/L KCl, 10.1 mmol/L Na_2_HPO_4_, 1.8 mmol/L KH_2_PO_4_, pH 7.4) containing 0.2% (w/v) LB broth mix were diluted 1:25 in the same medium and grown to an OD_600 nm_ = 0.1. Subcultures were diluted 1:500 in PBS/LB medium and 150 *μ*L was added to triplicate wells of a flat‐bottom 96‐well polystyrene plate. Plates were covered with 96‐well pin lids (Nalge Nunc International, Rochester, NY) and sealed with parafilm. Following 19 h incubation at 37°C and 200 rpm, pin lids were submerged in PBS without shaking for 10 min to remove loosely adhering bacteria, then stained for 15 min with 200 *μ*L 0.1% (w/v) crystal violet (CV) in 96‐well plates. Following CV staining, pin lids were washed 5 × 15 min in sterile water. CV was solubilized in 200 *μ*L of 33.3% acetic acid per well for 5 min in 96‐well plates and quantified by measuring absorbance at 600 nm. To rule out growth rate‐related effects on biofilm production, planktonic growth was also measured at the end of the 19 h assay at OD_600 nm_ and all strains reached the same terminal OD. Assays were performed three times independently and statistical significance was calculated using a two‐tailed Student's *t*‐test. A *P *≤ 0.05 was considered statistically significant.

### Time–kill assays

Overnight bacterial cultures in MHB were subcultured 1:50 in the same medium and grown to logarithmic phase at 37°C, with shaking at 200 rpm. Cultures were standardized to an OD_600 nm_ = 0.1 and diluted 1:200 in MHB. Aliquots of 1 mL were treated with and without piperacillin (12 *μ*g/mL final concentration) and incubated at 37°C for up to 24 h. Cultures were then serially diluted in MHB followed by spreading 50 *μ*L on MHA and incubated overnight at 37°C. The survival of strains treated with piperacillin was determined by enumerating CFUs from antibiotic‐treated samples compared to matched, untreated samples. Data were plotted as a log_10_ reduction in CFUs. Time–kill assays were performed three times independently and statistical significance was assessed using a two‐tailed Student's *t*‐test. A *P *≤ 0.05 was considered statistically significant.

## Results

### Loss of specific LTs decreases *β*‐lactam sensitivity

To understand the potential of LTs to modulate *β*‐lactam sensitivity in *P. aeruginosa*, single or multiple LT genes were deleted. An antibiotic sensitivity screen of single mutants revealed six with altered sensitivities to piperacillin, cefotaxime, and ceftazidime (Table 2). Loss of *sltB1* increased piperacillin and cefotaxime MICs ~twofold in Etest and broth microdilution assays. Loss of *mltB* increased piperacillin MICs by twofold using Etest; however, there was no increase in MIC using broth microdilution. Loss of *mltB* increased cefotaxime MICs by ≥twofold as confirmed by both methods. Loss of *sltH* resulted in a ≥twofold increase in piperacillin and cefotaxime MICs by Etest and broth microdilution. Mutants lacking *mltD* and *mltF2* had piperacillin MICs twofold higher than wild type as determined by Etest; however, the broth microdilution method showed no increase. Loss of *mltD* and *mltF2* also increased cefotaxime MICs by ≥twofold by both methods. In contrast, loss of *slt* resulted in a two to fourfold decrease in MICs of piperacillin, cefotaxime, and ceftazidime using Etest and broth microdilution methods, consistent with a previous report (Cavallari et al. 2013). Loss of *sltG*,* mltA*, or *mltF* had no effect on *β*‐lactam sensitivity (refer to Table S2 for a summary of MICs). Single mutants had wild‐type sensitivities to non‐*β*‐lactam antibiotics ciprofloxacin and vancomycin (Table 2), suggesting that MIC changes were not due to differences in efflux or membrane permeability. There were no differences between single LT mutants and the wild type in morphology (not shown) or growth rates over 48 h (Fig. S1), ruling out growth rate‐related changes in sensitivity.

To relate the mutants' *β*‐lactam MICs to AmpC expression, AmpC levels in uninduced and cefoxitin‐induced cultures were examined. No differences in AmpC expression or induction were detected for single LT mutants (Fig. S2). The MICs of double mutants lacking LTs and *ampC* were also determined (Table 2). While the loss of AmpC in LT mutant backgrounds reduced piperacillin and cefotaxime MICs, resistance levels remained above those of the *ampC* control. Thus, the data suggest that additional mechanism(s) – beyond AmpC expression – contributed to the increased MICs.

We showed previously by complementation with active‐site point mutants that loss of SltB1 activity – rather than potential disruption of protein complexes containing SltB1 – increased *β*‐lactam MICs (Cavallari et al. 2013). To determine whether this was true for other LT mutants with altered MICs, the *mltB*,* mltD*, and *slt* genes were replaced at their native loci with versions encoding putative active‐site mutants. The catalytic Glu162 of MltB (van Asselt et al. 1999; Blackburn and Clarke 2001), Glu144 of MltD (Blackburn and Clarke 2001), and Glu503 of Slt (Thunnissen et al. 1994; Blackburn and Clarke 2001) were each replaced with Ala. The *mltB* and *mltD* point mutants had wild‐type MICs (Table 2), suggesting a structural rather than enzymatic contribution of their protein products to *β*‐lactam sensitivity. In contrast, the Slt active‐site mutant failed to restore wild‐type *β*‐lactam MICs, suggesting that loss of Slt activity underlies enhanced sensitivity of the deletion mutant.

### The phenotypes of LT combination mutants correlate with loss of specific enzymes

**Figure 1:**
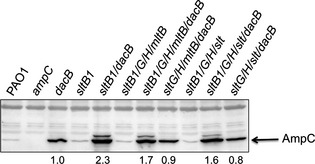
Only loss of *sltB1* further increases AmpC *β*‐lactamase expression in the *dacB* background. Loss of *dacB* in strains lacking all sLTs or Family 3 LTs increased AmpC levels, but not to the same level as the *dacB sltB1* double mutant. AmpC expression was reverted to that of the *dacB* single mutant when *sltB1* is present. Shown is a representative AmpC *β*‐lactamase immunoblot. Values represent average AmpC levels (*N* = 3), relative to the *dacB* mutant. sLTs, soluble lytic transglycosylases.

Similar to the way in which *β*‐lactams can target multiple PBPs (Kocaoglu and Carlson 2015; Kocaoglu et al. 2015), a small molecule inhibitor of LTs could simultaneously inactivate multiple enzymes due to the conservation of the LT active site (Scheurwater et al. 2008). To examine the effect of coinactivating multiple LTs on *β*‐lactam sensitivity, combination mutants lacking up to five LTs were generated (Table 1). The strategy for generation of combination mutants was based on a previous report in which LTs were divided into four families (Blackburn and Clarke 2001). We made combination mutants lacking all mLTs (i.e., *mltA*,* mltB*,* mltD*,* mltF*, and *mltF2*), all Family 1 LTs (i.e., *mltD*,* mltF*,* mltF2*, and *slt*), all sLTs (i.e., *sltB1*,* sltG*,* sltH*, and *slt)*, or all Family 3 LTs (i.e., *sltB1*,* sltG*,* sltH*, and *mltB*) (Table 1). With the exception of Slt, all Family 1 LTs are membrane‐bound enzymes, and with the exception of MltB, all Family 3 LTs are soluble enzymes. Two different phenotypes resulted, depending on the nature of the combinations. The *sltB1 mltB* double mutant had the highest *β*‐lactam MICs among combination mutants, with a ≥fourfold increase in piperacillin and cefotaxime MIC compared to wild type (Table 2). We confirmed by anti‐AmpC western blot (data not shown) that this increase in MIC was not due to increased AmpC levels in the *sltB1 mltB* mutant (Cavallari et al. 2013). Other combination mutants lacking sLTs or Family 3 LTs had increased *β*‐lactam MICs compared to wild type; however, none were as high as the *sltB1 mltB* double mutant. Changes to *β*‐lactam sensitivities were independent of changes in AmpC levels, which remained similar to wild type (Fig. 1) (Cavallari et al. 2013). No differences in ciprofloxacin or vancomycin MICs for any of the sLT or Family 3 combination mutants were observed. To test whether loss of Slt in the *β*‐lactam‐resistant *dacB* background could restore *β*‐lactam sensitivity, a *dacB slt* double mutant was generated. *β*‐lactam MICs were reduced at least twofold in the *dacB slt* double mutant compared to its *dacB* parent (Table 2).

The loss of all mLTs (i.e., *mltA*/*B*/*D*/*F*/*F2*) or Family 1 LTs (i.e., *mltD*/*F*/*F2*/*slt*) reduced *β*‐lactam MICs below wild‐type levels (Table 2). The *mltB*/*D*/*F* and *mltB*/*F*/*F2* triple mutants had cefotaxime MICs twofold below wild type. These strains lack *mltB –* which alone, increased cefotaxime MICs. The *mltD*/*F*/*F2* triple mutant and the *mltB*/*D*/*F*/*F2* quadruple mutant had twofold reductions in piperacillin and cefotaxime MICs compared to wild type, as confirmed by Etest and broth microdilution methods. The Family 1 LT mutant lacking *mltD*/*F*/*F2*/*slt* was highly sensitive to *β*‐lactams, with ≥fourfold decreases in piperacillin and cefotaxime MICs, respectively. This mutant was also ≥twofold more sensitive to ceftazidime and imipenem. The *mltA*/*B*/*D*/*F*/*F2* mutant lacking all mLTs was ≥three and ≥twofold more sensitive to piperacillin and cefotaxime, respectively, as confirmed by Etest and broth microdilution methods. Surprisingly, the *mltD*/*F*/*F2*,* mltB*/*D*/*F*/*F2*,* mltD*/*F*/*F2*/*slt* and *mltA*/*B*/*D*/*F*/*F2* mutants had vancomycin MICs that were 4‐, 8‐, 8‐, and 16‐fold lower than wild type, respectively, as determined by broth microdilution (Table 2). Vancomycin is normally incapable of crossing the OM, suggesting that increased drug sensitivities of combination mLT mutants were due in part to cell envelope perturbations.

### Only the loss of SltB1 – but not other sLTs or Family 3 LTs – increases AmpC expression in the *dacB* background

PBP4 (*dacB*) mutants of *P. aeruginosa* express AmpC at high levels, even in the absence of antibiotics (Moya et al. 2009; Cavallari et al. 2013). We showed previously that the combined loss of *sltB1* and *dacB* further increases AmpC expression, even though an *sltB1* mutant had wild‐type AmpC expression (Cavallari et al. 2013). Thus, we asked whether AmpC expression in the *dacB* background was altered by the loss of other sLTs or Family 3 LTs. A strain lacking all Family 3 LTs plus *dacB* (i.e., the *sltB1*/*G*/*H*/*mltB*/*dacB* mutant) had higher levels of AmpC than the *dacB* mutant alone (*P* < 0.01), but lower than the *sltB1 dacB* double mutant (*P* < 0.01; Fig. 1, compare lanes 5 and 7). To test whether the elevated AmpC levels in the *sltB1*/*G*/*H*/*mltB*/*dacB* mutant were due to the loss of SltB1, the disrupted *sltB1* gene was replaced with the wild‐type *sltB1* allele, yielding a *sltG*/*H*/*mltB*/*dacB* mutant. This mutant's AmpC expression was similar to that of the *dacB* single mutant (*P* = 0.97) (Fig. 1, compare lanes 7 and 8). Similarly, a strain lacking all sLTs (*sltB1*/*G*/*H*/*slt*) and *dacB* expressed more AmpC than the *dacB* mutant (*P* = 0.02), but less than the *sltB1 dacB* double mutant (*P* < 0.01; Fig. 1, compare lanes 5 and 10). Knock‐in of the wild‐type *sltB1* allele in this mutant – yielding the *sltG*/*H*/*slt*/*dacB* mutant – reversed AmpC levels to those of a *dacB* single mutant (*P* = 0.2) (Fig. 1, compare lanes 10 and 11). These data suggest that only loss of SltB1 increased AmpC expression in the *dacB* background. Furthermore, the loss of additional LTs in a strain lacking both PBP4 and SltB1 reduces its very high AmpC levels; however, the cause(s) of the attenuated AmpC levels in these strains is unknown.

**Figure 2:**
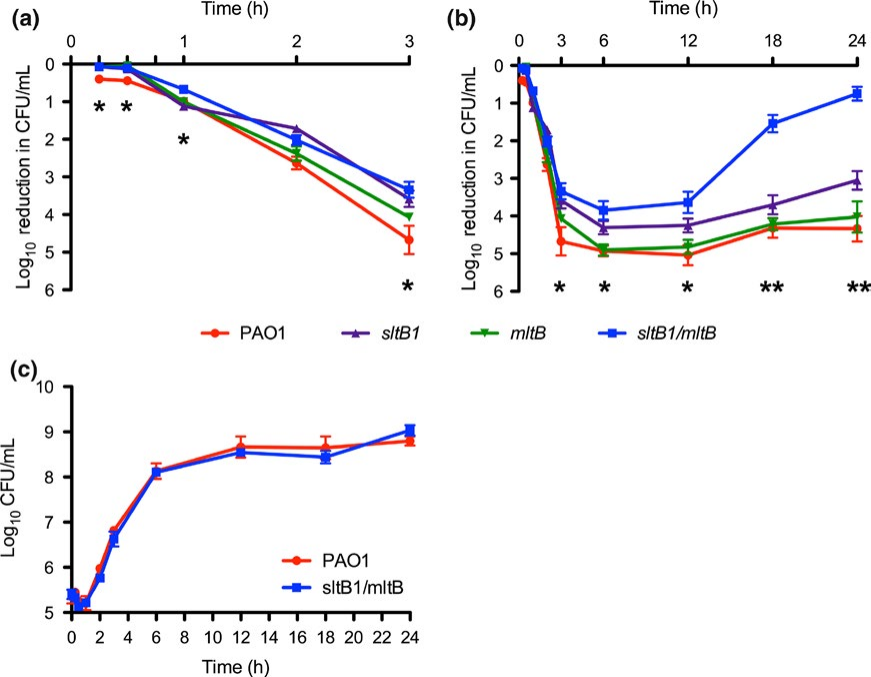
Strains lacking SltB1 and MltB die more slowly than wild‐type cells when treated with piperacillin. LT mutant cells were treated with piperacillin and their viability over 24 h was compared to untreated cells. Loss of *sltB1*/*mltB* slows piperacillin‐induced death over the first 3 h (A) with the remaining cells regrowing over 24 h (B). The growth rates between PAO1 and *sltB1*/*mltB* are the same (C), ruling out growth‐related differences in MIC. Asterisks indicate statistical differences between *sltB1*/*mltB* and PAO1. *N* = 3. Bars represent the mean ± SEM. *P <* *0.05; **P <* *0.01. LT, lytic transglycosylase; MIC, minimum inhibitory concentration.

Previous studies suggest that the loss of lytic enzymes slows *β*‐lactam‐induced cell death due to decreased cell lysis upon the inhibition of synthetic enzymes (Tomasz et al. 1970; Tomasz 1979; Heidrich et al. 2002; Uehara et al. 2009). This phenomenon has been referred to as penicillin tolerance (Tomasz et al. 1970; Tomasz 1979). To test whether slower rates of death could explain why mutants lacking Family 3 LTs had higher *β*‐lactam MICs despite wild‐type AmpC levels (Fig. 1, Fig. S2; Cavallari et al. 2013), piperacillin time–kill assays were performed (Fig. 2). Within 15 min, there was a ~0.4 log reduction in wild‐type CFU versus ~0.07 log (*P* = 0.02) for *sltB1*,* mltB*, and *sltB1 mltB* mutants (Fig. 2A). The reduced rate of killing by piperacillin in the LT mutants continued throughout the first 3 h, with a ~4.5‐log reduction in wild‐type CFU compared to ~3.5‐log and ~3‐log reductions for *sltB1* (*P* < 0.01) and *sltB1 mltB* (*P* < 0.001) mutants, respectively. After 6 h, cell death leveled off in all strains (Fig. 2B). Marginal regrowth was observed in wild‐type and single mutants between 6 and 24 h, whereas the *sltB1 mltB* mutant regrew to nearly the same concentration as the untreated cells. The wild‐type and *sltB1 mltB* strains had indistinguishable growth curves under the assay conditions, ruling out growth rate‐related differences in MIC (Fig. 2C). These data suggest that the elevated *β*‐lactam MICs for some LT mutants is due in part to a reduced rate of autolysis.

### Combination mLT mutants have compromised cell envelopes

**Figure 3:**
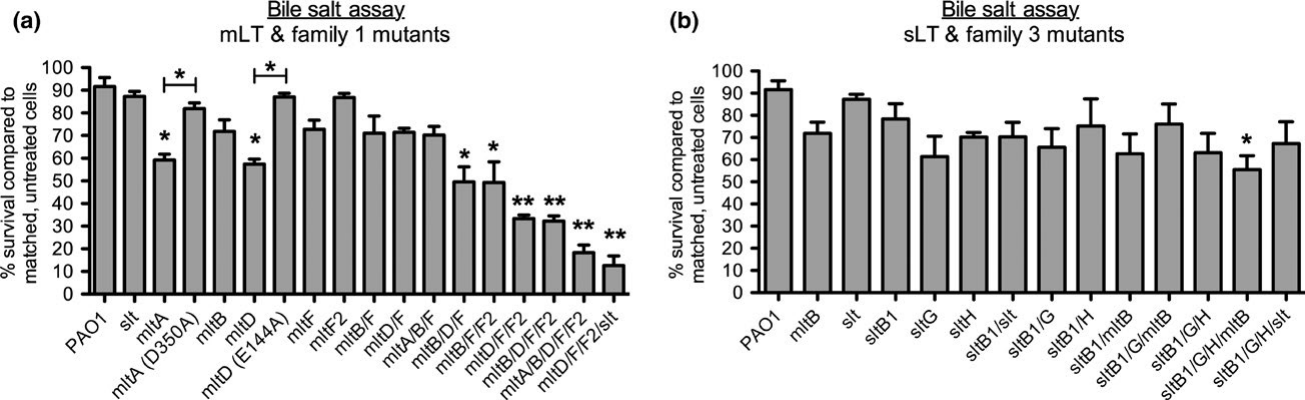
Combination mLT and Family 1 LT mutants have increased OM permeability. Viability of LT mutants grown in the presence/absence of bile salts was compared. (A) mLT and Family 1 mutant strains were sensitive to bile salts while (B) sLT and Family 3 mutants were not. *N* = 3. Bars represent the mean ± SEM. *P <* *0.05; **P <* *0.01. mLT, membrane‐bound lytic transglycosylase; OM, outer membrane; sLT, soluble lytic transglycosylase.

Gram‐negative bacteria with compromised OMs are sensitive to detergent‐like compounds, including bile salts (Hancock 1984; Lamers et al. 2013). Due to their marked increase in vancomycin susceptibility, we further tested mutants lacking Family 1 and mLT enzymes for increased OM permeability using bile salt sensitivity assays (Fig. 3). Of the single LT mutants, only *mltA* (*P* = 0.03) and *mltD* (*P* = 0.02) were more sensitive than wild type (Fig. 3A). Chromosomal replacement of *mltA* and *mltD* with versions encoding active‐site mutants MltA D305A (van Straaten et al. 2005; Powell et al. 2006) and MltD E144A (Blackburn and Clarke 2001) restored bile salt resistance to near‐wild‐type levels (Fig. 3A). Mutants lacking Family 3 LTs or all sLTs were unaffected by bile salts, with the exception of the *sltB1*/*G*/*H*/*mltB* quadruple mutant (~55% of wild‐type viability; *P* = 0.018) (Fig. 3B). Conversely, mutants lacking all Family 1 LTs or all mLTs were more sensitive than wild type. Those mutants most sensitive to *β*‐lactams – *mltD*/*F*/*F2*,* mltD*/*F*/*F2*/*slt*,* mltB*/*D*/*F*/*F2*, and *mltA*/*B*/*D*/*F*/*F2* – had ~15–30% survival rates on bile salts compared to matched, untreated cells (Fig. 3A). Triple mutants *mltB*/*D*/*F* and *mltB*/*F*/*F2* had survival rates of ~50% (*P* = 0.01 and 0.02, respectively).

Loss of lipopolysaccharide (LPS) integrity can increase OM permeability (Hancock 1984; Nikaido 2003; Sutterlin et al. 2014); however, supplementation with 1 mmol/L MgCl_2_ (Plesiat et al. 1997; Nikaido 2003; Lamers et al. 2013) had no effect on the sensitivity of *mltD*/*F*/*F2*/*slt* or *mltA*/*B*/*D*/*F*/*F2* mutants to bile salts (Fig. S3), suggesting that increased OM permeability was independent of LPS stability. To further establish whether OM permeability was related to LPS instability, mLT mutants were assessed for sensitivity to the antimicrobial peptide polymyxin B (PMB). The potency of PMB is directly affected by LPS integrity and requires the negative charge to access the periplasm (Delcour 2009). PMB sensitivity was generally unaffected by the loss of mLTs except for a twofold reduction in MIC for *mltB*/*D*/*F*/*F2*,* mltD*/*F*/*F2*/*slt*, or *mltA*/*B*/*D*/*F*/*F2* mutants (0.5 *μ*g/mL) compared to wild type (1 *μ*g/mL) (Table 2) – further suggesting that the increase in OM permeability is largely independent of LPS stability.

**Figure 4:**
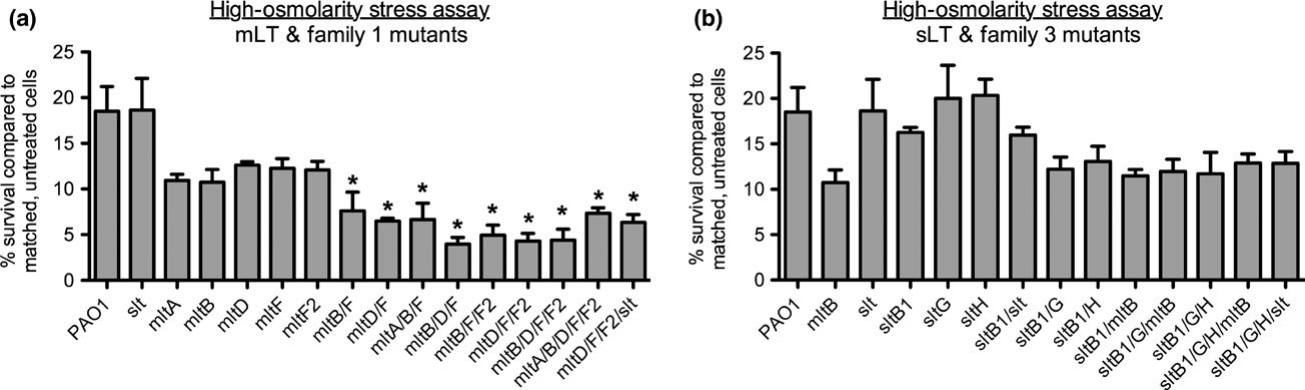
Combination mLT and Family 1 LT mutants are sensitive to osmotic stress. Viability of LT mutants was compared between cells treated with 2.5 mol/L NaCl and untreated, control cells. (A) mLT and Family 1 mutant strains were more sensitive to osmotic stress than wild type while (B) sLT and Family 3 mutants were not. *N* = 3. Bars represent the mean ± SEM. *P <* *0.05. mLT, membrane‐bound lytic transglycosylase; sLT, soluble lytic transglycosylase.

The integrity of the PG layer in cells lacking hydrolases can be investigated by osmotic stress challenge, by growth on media containing 2.5 mol/L NaCl (Heidrich et al. 2002; Korsak et al. 2005). Single mLT mutants lacking *mltA*,* mltB*,* mltD*,* mltF*, or *mltF2* (Fig. 4A) and single sLT mutants lacking *slt*,* sltB1*,* sltG*, or *sltH* (Fig. 4B) had wild‐type survival on high‐salt medium. While combination sLT and Family 3 LT mutants had wild‐type levels of survival on high salt (Fig. 4B), combination mLT and Family 1 LT mutants lacking two or more enzymes had statistically significant reductions in viability (Fig. 4A). The sensitivity of combination mLT mutants – but not combination sLT mutants – to osmotic stress supports a role for mLTs in cell wall integrity.

### Complementation of mLT mutants with Lpp reduces sensitivity to bile salts

**Figure 5:**
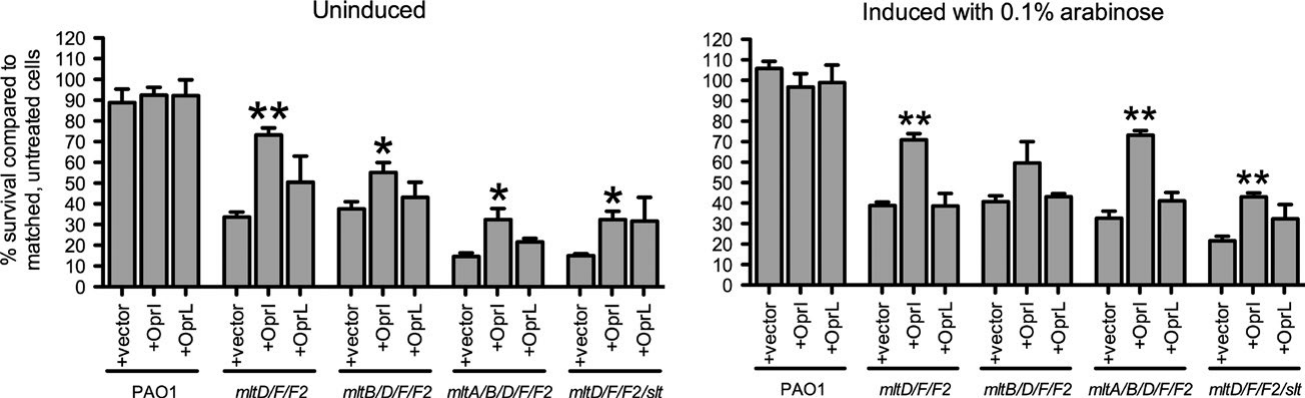
Overexpression of Braun's lipoprotein in trans reduces sensitivity to bile salts in membrane‐bound lytic transglycosylase (mLT) mutants. mLT mutants sensitive to bile salts were complemented with OprI (Braun's lipoprotein; Lpp) or OprL (peptidoglycan‐associated lipoprotein; Pal) and their viability in the presence of bile salts was assessed. Left panel, uninduced; right panel, induced with 0.1% arabinose. *N* = 3. Bars represent the mean ± SEM. *P <* *0.05; **P <* *0.01.

Based on the phenotypes of multi‐mLT mutants, we hypothesized that these lipoproteins might help to tether the OM to PG, similar to Lpp (called OprI or PA2853 in *P. aeruginosa*) (Mizuno and Kageyama 1979; Cornelis et al. 1989; Cascales et al. 2002; Ni and Chen 2004, 2005; Wessel et al. 2013) and PG‐associated lipoprotein, Pal (called OprL or PA0973) (Mizuno 1979; Mizuno and Kageyama 1979; Hancock et al. 1981; Cascales et al. 2002). To test whether increased OM–PG interactions might improve the mLT mutants' cell envelope integrity, OprI or OprL was expressed in trans in strains sensitive to bile salts (Fig. 5). Low‐level expression of OprI from the vector's leaky promoter in the absence of induction (Giltner et al. 2010) increased survival on bile salts from ~34% to ~73% for the *mltD*/*F*/*F2* mutant and for strains lacking all mLTs (*mltA*/*B*/*D*/*F*/*F2*), or all Family 1 LTs (*mltD*/*F*/*F2*/*slt*), from ~14% to ~32% (Fig. 5, left panel). Using 0.1% l‐arabinose to increase OprI expression did not further improve bile salt survival (Fig. 5, right panel). Complementation with OprL, with or without l‐arabinose induction, had no significant effect on bile salt sensitivity of any mutants tested.

### Loss of mLTs increases biofilm formation

**Figure 6:**
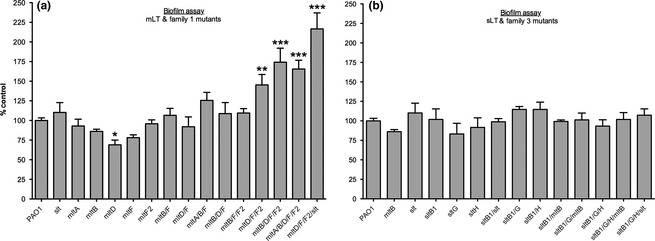
Combination mutants lacking mLTs or Family 1 LTs are hyperbiofilm formers. Biofilms of LT mutants grown for 24 h were compared to those formed by wild type. (A) Loss of multiple mLTs increased biofilm formation while (B) the loss of sLTs did not. *N* = 3. Bars represent the mean ± SEM. *P <* *0.05; **P <* *0.01; ***P <* *0.001. mLT, membrane‐bound lytic transglycosylase; sLT, soluble lytic transglycosylase.

LT activity, cell wall turnover, and cell envelope damage have previously been linked to increased biofilm formation in both Gram‐negative (Monteiro et al. 2011) and Gram‐positive bacteria (Sailer et al. 2003; Kolar et al. 2011). Moreover, exposure to sub‐MIC *β*‐lactams can induce biofilm formation in an autolysin‐dependent manner (Kaplan et al. 2012). Cells in a biofilm are protected from exposure to many antibiotics by the surrounding extracellular polymeric substance matrix and are heterogeneous with respect to growth rates, with reduced sensitivity in slow‐growing or persister subpopulations (Kostakioti et al. 2013). Factors affecting cell wall metabolism can modulate bacterial surface properties (Reid et al. 2004; Liu et al. 2012) and biofilm formation (Monteiro et al. 2011; Payne et al. 2013; Brambilla et al. 2014), altering adhesion to surfaces or the ability to form the protective matrix (Monteiro et al. 2011; Liu et al. 2014). Therefore, we asked whether the ability of LT mutants to form biofilms was altered. Only the *mltD* mutant made less biofilm than wild type (~70% of wild type; *P* = 0.03), while four mutants – the *mltD*/*F*/*F2* mutant and its derivatives *mltD*/*F*/*F2*/*slt*,* mltB*/*D*/*F*/*F2*, and *mltA*/*B*/*D*/*F*/*F2*, all of which have compromised cell envelopes based on the data presented above – showed significantly enhanced biofilm formation (Fig. 6). The parent strain of *mltD*/*F*/*F2*,* mltD*/*F*, made wild‐type levels of biofilm. The *mltA*/*B*/*F* and *mltB*/*D*/*F* triple mutants had elevated biofilm production compared with their *mltB*/*F* parent strain, which resembled wild type (Fig. 6).

## Discussion

Resistance to antibiotics has increased dramatically, and the need to identify novel targets is urgent (World Health Organization, 2014). PG is essential for bacterial survival and absent in eukaryotes, making it a favored target for antibacterial agents. Previous profiling of the *β*‐lactam sensitivities of *P. aeruginosa* mutants lacking PG‐active enzymes showed that loss of specific LTs – which have been proposed as potential drug targets (Blackburn and Clarke 2001) – had varying effects on resistance (Cavallari et al. 2013). Our work, and that of others (Kraft et al. 1999; Korsak et al. 2005), have questioned the degree of redundancy among LTs, and prompted this more comprehensive study.

We identified five LT deletion mutants with increased *β*‐lactam MICs (i.e., those lacking SltB1, SltH, MltB, MltD, and MltF2), and one with decreased MICs (i.e., that lacking Slt; summarized in Table S3). Consistent with a previous study of transposon mutants (Cavallari et al. 2013), loss of SltB1 or MltB increased MICs, while loss of Slt decreased MICs. In contrast, loss of MltF had no effect on sensitivity in the current study. These differences may relate to the use of transposon mutants for the previous study versus clean gene deletions for this work. We have now identified three additional LTs – SltH, MltD, and MltF2 – whose absence led to elevated *β*‐lactam MICs. Loss of AmpC in these strains reduced *β*‐lactam MICs, but they remained higher than the *ampC* control. These data suggest that AmpC‐independent mechanisms contribute to the reduced sensitivity of LT mutants.

As has been observed for cells lacking multiple PG hydrolases (Tomasz et al. 1970; Tomasz 1979; Heidrich et al. 2002; Uehara et al. 2009), the loss of LTs could be protective to cells challenged with *β*‐lactams, by limiting autolysis under conditions where PG biosynthesis is inhibited – the antisuicide hypothesis (Cavallari et al. 2013). Supporting this idea, cells lacking SltB1 and MltB had slower rates of cell death compared to wild‐type cells when treated with piperacillin (Fig. 2). Loss of additional LTs did not exacerbate the phenotype, possibly because only a subset of LTs is active at certain times during the cell cycle, or because different LTs make specific contributions to PG turnover (Blackburn and Clarke 2002). Blocking LT activity with the inhibitor NAG‐thiazoline (Reid et al. 2004) or deleting a combination of specific LTs and amidases (Heidrich et al. 2002) significantly reduced levels of ampicillin‐induced *E. coli* cell lysis, providing further evidence that loss of lytic activities can prolong cell survival in the presence of PG‐synthesis inhibitors.

Slt is a unique exception to this phenomenon. In *E. coli*, loss of Slt reduces *β*‐lactam resistance and prevents induction of AmpC from a plasmid carrying *Enterobacter cloacae ampR* and *ampC* (Templin et al. 1992; Kraft et al. 1999; Cho et al. 2014). In *P. aeruginosa*, Slt was the only LT whose loss increased *β*‐lactam sensitivity (Table S3), although AmpC levels were unchanged (this study; Cavallari et al. 2013). The critical contribution of *E. coli* Slt to *β*‐lactam susceptibility was recently attributed to its role in a futile cycle of nascent PG synthesis and degradation upon *β*‐lactam challenge (Cho et al. 2014). The simultaneous loss of Slt and *β*‐lactam challenge was hypothesized to result in the accumulation of aberrantly cross‐linked glycan strands in the mature PG matrix that interfered with the function of downstream PG‐remodeling machineries (Cho et al. 2014). Whether this paradigm holds true for *P. aeruginosa* and other Gram‐negatives will be an important future consideration, particularly given the differences between species in the number of LTs and phenotypes of deletion mutants.

While the loss of Family 3 LTs or sLTs increased *β*‐lactam MICs, the loss of mLTs or all Family 1 LTs caused extreme *β*‐lactam sensitivity, as well as sensitivity to detergent‐like bile salts and to vancomycin, consistent with a compromised OM (compare *sltB1*/*G*/*H*/*mltB* and *sltB1*/*G*/*H*/*slt* strains to *mltA*/*B*/*D*/*F*/*F2* and *mltD*/*F*/*F2*/*slt* in summary Table S3). Strains lacking multiple sLTs had wild‐type sensitivity to bile salts – except for the mutant lacking all Family 3 LTs, which includes MltB – suggesting that only mLTs contribute to OM stability. Combination mLT mutants were also sensitive to osmotic stress – suggestive of perturbations to the PG layer – implying that they suffer from general cell envelope defects (refer to Table S3, strains *mltA*/*B*/*D*/*F*/*F2* and *mltD*/*F*/*F2*/*slt*). Membrane defects were unlikely to be due to LPS perturbation, since PMB sensitivity was largely unchanged in mLT mutants and Mg^2+^ supplementation failed to reverse bile salt sensitivity. Furthermore, complementation with MltA and MltD active‐site point mutants restored wild‐type sensitivity to bile salts (Fig. 3A), pointing to a structural role for these proteins – possibly by facilitating the formation or functioning of multiprotein complexes or by stabilizing OM–PG interactions. In contrast, Slt (this study) and SltB1 (Cavallari et al. 2013) active‐site mutants phenocopied their respective deletion mutants (Table S3), suggesting that catalytic activity of sLTs is essential. The specific roles of most LTs in *P. aeruginosa* remain unknown (Lee et al. 2015), and future studies to further delineate functional differences between sLTs and mLTs are necessary.

mLTs are OM lipoproteins (Derouaux et al. 2014) that could contribute to stabilizing OM–PG interactions. There are multiple examples in Gram‐negative bacteria of PG‐binding lipoproteins that are critical for OM barrier function. These include Lpp (Cascales et al. 2002; Ni and Chen 2004, 2005), called OprI in *P. aeruginosa* (Mizuno and Kageyama 1979; Wessel et al. 2013), and Pal (Cascales et al. 2002), called OprL (Mizuno 1979; Mizuno and Kageyama 1979; Hancock et al. 1981). Lpp is the most abundant Pal in *E. coli*, with ~33% covalently bound to PG through the DAP moiety of PG stem peptides (Neidhardt et al. 1990; Ni and Chen 2005). OprI is also abundant in *P. aeruginosa*, accounting for ~20% of total OM proteins (Mizuno and Kageyama 1979; Cornelis et al. 1989), but exhibits strain‐specific variations in the level of covalent PG attachment (Mizuno and Kageyama 1979; Hancock et al. 1981; Duchene et al. 1989; Wessel et al. 2013). Pal is less abundant in *E. coli* than Lpp – 2000–3000 molecules per *μ*m^2^ versus ~100,000 molecules per *μ*m^2^ for Lpp (Nikaido 1996; Cascales et al. 2002) – but its absence causes similar perturbations of the cell envelope, suggesting that factors in addition to protein density determine the relative contributions of these proteins to cell envelope integrity. Similarly, loss of covalent OM–PG interactions is not solely responsible for cell envelope instability in *lpp* mutants, since overexpression of Pal in that background restored membrane integrity, while the reverse was not true (Cascales et al. 2002).

Reversal of bile salt sensitivity in multi‐mLT mutants complemented with OprI suggested that in addition to any enzymatic roles they might have, mLTs might help to stabilize OM–PG interactions. The inability of OprL to reverse bile salt sensitivity was unexpected, given Pal's ability to stabilize the OM in Lpp‐deficient *E. coli*; however, these data are consistent with a recent study (Wessel et al. 2013) investigating *P. aeruginosa* OM vesicle (OMV) production and OM stability. Loss of OprI increased OMV production in *P. aeruginosa* – presumably due to decreased OM–PG interactions – while loss of OprL had no effect (Wessel et al. 2013). Thus, the roles of *P. aeruginosa* Lpp and Pal orthologs in OM stability appear different from those in *E. coli*. However, we cannot rule out the possibility that overexpression of OprI suppressed the effects of the mLT mutants by stabilizing a cell envelope perturbed via a different mechanism.

Establishing the mechanisms underlying changes in biofilm formation in PG remodeling enzyme mutants has been challenging due to the lack of a consistent phenotype when such enzymes are lost (Gallant et al. 2005; Monteiro et al. 2011; Payne et al. 2013; Brambilla et al. 2014). Only the *mltD* mutant in this study was defective for biofilm formation, while four strains lacking mLTs and Family 1 LTs produced significantly more biofilm. These four strains have compromised OMs and increased sensitivity to osmotic stress (Table S3). Loss of OM integrity in these mutants may promote biofilm formation by invoking a response to perceived cell envelope damage. Such a response is reminiscent of the triggering of AmpC expression that occurs – even in the absence of antibiotics – upon loss of PBP4 (Moya et al. 2009).

Our previous study (Cavallari et al. 2013) showed that loss of LTs from Family 1 reduced *β*‐lactam MICs while loss of LTs from Family 3 increased MICs; however, in light of more comprehensive data, it is clear that differences in *β*‐lactam MICs are more accurately related to the loss of soluble versus membrane‐bound enzymes (Table S3). Our data agree with previous studies (Korsak et al. 2005; Lee et al. 2013) that there is a degree of functional redundancy between LTs, in that (1) multiple enzymes can be deleted without lethality and (2) with the exception of MltA and MltD, multiple enzymes must be deleted before changes to cell wall integrity are observed (Table S3). Furthermore, AmpC induction was unaffected by the loss of multiple LTs, suggesting redundancy in generating activating anhMP fragments. In contrast, inactivation of *E. coli* Slt, MltA or MltB attenuated AmpC induction (Kraft et al. 1999), implying that those enzymes make unique contributions to the pool of activating anhMPs. Mecillinam treatment of an *E. coli slt* mutant resulted in a different PG profile than that of wild type challenged with the same drug (Cho et al. 2014), suggesting that Slt has a specific role in muropeptide turnover upon *β*‐lactam challenge. In *P. aeruginosa*, only a subset of LTs affects *β*‐lactam sensitivity, and only the absence of *sltB1* – but not other sLTs or *mltB* – uniquely contributes to further increases in AmpC levels when PBP4 is inactivated. Thus, the repertoire of LTs with unique activities differs between species.

A screen of the *P. aeruginosa* strain PA14 transposon mutant library identified several genes whose disruption altered *β*‐lactam MICs; however, none encoded LTs (Alvarez‐Ortega et al. 2010). The use of clean deletion mutants – or the PAO1 background strain – in this study may underlie the differences in our results. Furthermore, combination mutants often reveal phenotypes that are not obvious from single mutant screens, a concept widely exploited in synthetic lethality studies (Bender and Pringle 1991; Bernhardt and de Boer 2004; Butland et al. 2008). Here, the combined loss of multiple mLTs was critical for uncovering their potential roles in cell envelope stability.

A thorough understanding of the roles of sLTs versus mLTs, and of the unique functions of specific LTs, is necessary for their further development as potential therapeutic targets. We showed that increased *β*‐lactam sensitivity of a mutant lacking all mLTs or all Family 1 LTs corresponds to loss of cell envelope integrity, while decreased *β*‐lactam sensitivity of sLT mutants is due – at least in part – to slower death. These findings suggest that pan‐inhibitors of LT activity could have the potential to increase *β*‐lactam resistance in *P. aeruginosa*, as – except for Slt – the activity of sLTs contributes to sensitivity, while mLT activity is dispensable. However, the collective inhibition of all LTs may yield unexpected results. As the sole LT in both *P. aeruginosa* and *E. coli* whose loss leads to increased *β*‐lactam sensitivity (Templin et al. 1992; Cavallari et al. 2013; Cho et al. 2014), Slt is emerging as the preferred LT target for development of antibiotic adjuvants.

## Conflict of Interest

None declared.

## Supporting information


**Table S1.** Primer details for PCR confirmation of LT mutant strains.
**Table S2.** MICs for all LT mutants of β‐lactam, fluoroquinolone, and glycopeptide antibiotics.
**Table S3.** Summary of phenotypes associated with LT mutants.
**Figure S1.** Loss of LTs does not cause growth defects. Bacterial growth (OD_600 nm_) was monitored every hour for 48 h using a plate‐based assay, described below. Curves are average of three biological replicates with three technical replicates each. Following the strain names in each legend are the growth rates per minute (min^−1^) and lag times (in min), respectively.
**Figure S2.** Loss of LTs does not affect AmpC expression. Strains lacking the indicated LT were grown under basal (top panel) or AmpC‐inducing (bottom panel) conditions and immunoblotted using α‐AmpC antibodies, as described in the Materials and Methods section. Loss of LTs does not prevent either basal AmpC expression or AmpC induction.
**Figure S3.** Magnesium supplementation does not affect bile salt sensitivity in mLT mutants. Bile salt assays were performed as described in the Materials and Methods section with and without MgCl_2_ (1 mmol/L final) supplementation. Bile salt sensitivity of the *mltA*/*B*/*D*/*F*/*F2* and *mltD*/*F*/*F2*/*slt* mutants was unaffected by the addition of magnesium. Bars representing non‐MgCl_2_‐treated samples are the same as those used in Figure 3A. *N* = 3. Bars represent the mean ± SEM.

## References

[mbo3286-bib-0001] Alvarez‐Ortega, C. , I. Wiegand , J. Olivares , R. E. Hancock , and J. L. Martinez . 2010 Genetic determinants involved in the susceptibility of *Pseudomonas aeruginosa* to beta‐lactam antibiotics. Antimicrob. Agents Chemother. 54:4159–4167. 20679510 10.1128/AAC.00257-10 PMC2944606

[mbo3286-bib-0002] Asikyan, M. L. , J. V. Kus , and L. L. Burrows . 2008 Novel proteins that modulate type IV pilus retraction dynamics in *Pseudomonas aeruginosa* . J. Bacteriol. 190:7022–7034. 18776014 10.1128/JB.00938-08 PMC2580705

[mbo3286-bib-0003] van Asselt, E. J. , A. J. Dijkstra , K. H. Kalk , B. Takacs , W. Keck , and B. W. Dijkstra . 1999 Crystal structure of *Escherichia coli* lytic transglycosylase Slt35 reveals a lysozyme‐like catalytic domain with an EF‐hand. Structure 7:1167–1180. 10545329 10.1016/s0969-2126(00)80051-9

[mbo3286-bib-0004] Bender, A. , and J. R. Pringle . 1991 Use of a screen for synthetic lethal and multicopy suppressee mutants to identify two new genes involved in morphogenesis in *Saccharomyces cerevisiae* . Mol. Cell. Biol. 11:1295–1305. 1996092 10.1128/mcb.11.3.1295-1305.1991 PMC369400

[mbo3286-bib-0005] Bernal, P. , C. Molina‐Santiago , A. Daddaoua , and M. A. Llamas . 2013 Antibiotic adjuvants: identification and clinical use. Microb. Biotechnol. 6:445–449. 23445397 10.1111/1751-7915.12044 PMC3918149

[mbo3286-bib-0006] Bernhardt, T. G. , and P. A. de Boer . 2004 Screening for synthetic lethal mutants in *Escherichia coli* and identification of EnvC (YibP) as a periplasmic septal ring factor with murein hydrolase activity. Mol. Microbiol. 52:1255–1269. 15165230 10.1111/j.1365-2958.2004.04063.x PMC4428336

[mbo3286-bib-0007] Blackburn, N. T. , and A. J. Clarke . 2001 Identification of four families of peptidoglycan lytic transglycosylases. J. Mol. Evol. 52:78–84. 11139297 10.1007/s002390010136

[mbo3286-bib-0008] Blackburn, N. T. , and A. J. Clarke . 2002 Characterization of soluble and membrane‐bound family 3 lytic transglycosylases from *Pseudomonas aeruginosa* . Biochemistry 41:1001–1013. 11790124 10.1021/bi011833k

[mbo3286-bib-0009] Boucher, H. W. , G. H. Talbot , J. S. Bradley , J. E. Edwards , D. Gilbert , L. B. Rice , et al. 2009 Bad bugs, no drugs: no ESKAPE! An update from the Infectious Diseases Society of America. Clin. Infect. Dis. 48:1–12. 19035777 10.1086/595011

[mbo3286-bib-0010] Brambilla, L. , J. Moran‐Barrio , and A. M. Viale . 2014 Low‐molecular‐mass penicillin binding protein 6b (DacD) is required for efficient GOB‐18 metallo‐beta‐lactamase biogenesis in *Salmonella enterica* and *Escherichia coli* . Antimicrob. Agents Chemother. 58:205–211. 24145538 10.1128/AAC.01224-13 PMC3910800

[mbo3286-bib-0011] Breidenstein, E. B. , C. De la Fuente‐Nunez , and R. E. Hancock . 2011 *Pseudomonas aeruginosa*: all roads lead to resistance. Trends Microbiol. 19:419–426. 21664819 10.1016/j.tim.2011.04.005

[mbo3286-bib-0012] Butland, G. , M. Babu , J. J. Diaz‐Mejia , F. Bohdana , S. Phanse , B. Gold , et al. 2008 eSGA: *E. coli* synthetic genetic array analysis. Nat. Methods 5:789–795. 18677321 10.1038/nmeth.1239

[mbo3286-bib-0013] Cascales, E. , A. Bernadac , M. Gavioli , J. C. Lazzaroni , and R. Lloubes . 2002 Pal lipoprotein of *Escherichia coli* plays a major role in outer membrane integrity. J. Bacteriol. 184:754–759. 11790745 10.1128/JB.184.3.754-759.2002 PMC139529

[mbo3286-bib-0014] Cavallari, J. F. , R. P. Lamers , E. M. Scheurwater , A. L. Matos , and L. L. Burrows . 2013 Changes to its peptidoglycan‐remodeling enzyme repertoire modulate beta‐lactam resistance in *Pseudomonas aeruginosa* . Antimicrob. Agents Chemother. 57:3078–3084. 23612194 10.1128/AAC.00268-13 PMC3697359

[mbo3286-bib-0015] Cho, H. , T. Uehara , and T. G. Bernhardt . 2014 Beta‐lactam antibiotics induce a lethal malfunctioning of the bacterial cell wall synthesis machinery. Cell 159:1300–1311. 25480295 10.1016/j.cell.2014.11.017 PMC4258230

[mbo3286-bib-0016] Cornelis, P. , A. Bouia , A. Belarbi , A. Guyonvarch , B. Kammerer , V. Hannaert , et al. 1989 Cloning and analysis of the gene for the major outer membrane lipoprotein from *Pseudomonas aeruginosa* . Mol. Microbiol. 3:421–428. 2473376 10.1111/j.1365-2958.1989.tb00187.x

[mbo3286-bib-0017] Davies, J. , and D. Davies . 2010 Origins and evolution of antibiotic resistance. Microbiol. Mol. Biol. Rev. 74:417–433. 20805405 10.1128/MMBR.00016-10 PMC2937522

[mbo3286-bib-0018] Delcour, A. H. 2009 Outer membrane permeability and antibiotic resistance. Biochim. Biophys. Acta 1794:808–816. 19100346 10.1016/j.bbapap.2008.11.005 PMC2696358

[mbo3286-bib-0019] Derouaux, A. , M. Terrak , T. Den blaauwen , and W. Vollmer . 2014 Bacterial cell wall growth, shape and division Pp. 3–54 *in* Remaut H. and Fronzes R., eds. Bacterial membranes: structural and molecular biology. Caister Academic Press, Norfolk, U.K.

[mbo3286-bib-0020] Duchene, M. , C. Barron , A. Schweizer , B. U. von Specht , and H. Domdey . 1989 *Pseudomonas aeruginosa* outer membrane lipoprotein I gene: molecular cloning, sequence, and expression in *Escherichia coli* . J. Bacteriol. 171:4130–4137. 2502533 10.1128/jb.171.8.4130-4137.1989 PMC210182

[mbo3286-bib-0021] Fernandez, L. , E. B. Breidenstein , and R. E. Hancock . 2011 Creeping baselines and adaptive resistance to antibiotics. Drug Resist. Updates 14:1–21. 10.1016/j.drup.2011.01.001 21288762

[mbo3286-bib-0022] Gallant, C. V. , C. Daniels , J. M. Leung , A. S. Ghosh , K. D. Young , L. P. Kotra , et al. 2005 Common beta‐lactamases inhibit bacterial biofilm formation. Mol. Microbiol. 58:1012–1024. 16262787 10.1111/j.1365-2958.2005.04892.x PMC3097517

[mbo3286-bib-0023] Giltner, C. L. , M. Habash , and L. L. Burrows . 2010 *Pseudomonas aeruginosa* minor pilins are incorporated into type IV pili. J. Mol. Biol. 398:444–461. 20338182 10.1016/j.jmb.2010.03.028

[mbo3286-bib-0024] Hancock, R. E. 1984 Alterations in outer membrane permeability. Annu. Rev. Microbiol. 38:237–264. 6093683 10.1146/annurev.mi.38.100184.001321

[mbo3286-bib-0025] Hancock, R. E. , R. T. Irvin , J. W. Costerton , and A. M. Carey . 1981 *Pseudomonas aeruginosa* outer membrane: peptidoglycan‐associated proteins. J. Bacteriol. 145:628–631. 6780523 10.1128/jb.145.1.628-631.1981 PMC217314

[mbo3286-bib-0026] Heidrich, C. , A. Ursinus , J. Berger , H. Schwarz , and J. V. Holtje . 2002 Effects of multiple deletions of murein hydrolases on viability, septum cleavage, and sensitivity to large toxic molecules in *Escherichia coli* . J. Bacteriol. 184:6093–6099. 12399477 10.1128/JB.184.22.6093-6099.2002 PMC151956

[mbo3286-bib-0027] Hoang, T. T. , R. R. Karkhoff‐Schweizer , A. J. Kutchma , and H. P. Schweizer . 1998 A broad‐host‐range Flp‐FRT recombination system for site‐specific excision of chromosomally‐located DNA sequences: application for isolation of unmarked *Pseudomonas aeruginosa* mutants. Gene 212:77–86. 9661666 10.1016/s0378-1119(98)00130-9

[mbo3286-bib-0028] Jacobs, C. , L. J. Huang , E. Bartowsky , S. Normark , and J. T. Park . 1994 Bacterial cell wall recycling provides cytosolic muropeptides as effectors for beta‐lactamase induction. EMBO J. 13:4684–4694. 7925310 10.1002/j.1460-2075.1994.tb06792.x PMC395403

[mbo3286-bib-0029] Johnson, J. W. , J. F. Fisher , and S. Mobashery . 2013 Bacterial cell‐wall recycling. Ann. N. Y. Acad. Sci. 1277:54–75. 23163477 10.1111/j.1749-6632.2012.06813.x PMC3556187

[mbo3286-bib-0030] Jorgenson, M. A. , Y. Chen , A. Yahashiri , D. L. Popham , and D. S. Weiss . 2014 The bacterial septal ring protein RlpA is a lytic transglycosylase that contributes to rod shape and daughter cell separation in *Pseudomonas aeruginosa* . Mol. Microbiol. 93:113–128. 24806796 10.1111/mmi.12643 PMC4086221

[mbo3286-bib-0031] Juan, C. , B. Moya , J. L. Perez , and A. Oliver . 2006 Stepwise upregulation of the *Pseudomonas aeruginosa* chromosomal cephalosporinase conferring high‐level beta‐lactam resistance involves three AmpD homologues. Antimicrob. Agents Chemother. 50:1780–1787. 16641450 10.1128/AAC.50.5.1780-1787.2006 PMC1472203

[mbo3286-bib-0032] Kaplan, J. B. , E. A. Izano , P. Gopal , M. T. Karwacki , S. Kim , J. L. Bose , et al. 2012 Low levels of beta‐lactam antibiotics induce extracellular DNA release and biofilm formation in *Staphylococcus aureus* . MBio 3:e00198‐12. 22851659 10.1128/mBio.00198-12 PMC3419523

[mbo3286-bib-0033] Kocaoglu, O. , and E. E. Carlson . 2015 Profiling of beta‐lactam selectivity for penicillin‐binding proteins in *Escherichia coli* strain DC2. Antimicrob. Agents Chemother. 59:2785–2790. 25733506 10.1128/AAC.04552-14 PMC4394777

[mbo3286-bib-0034] Kocaoglu, O. , H. C. Tsui , M. E. Winkler , and E. E. Carlson . 2015 Profiling of beta‐lactam selectivity for penicillin‐binding proteins in *Streptococcus pneumoniae* D39. Antimicrob. Agents Chemother. 59:3548–3555. 25845878 10.1128/AAC.05142-14 PMC4432181

[mbo3286-bib-0035] Kolar, S. L. , V. Nagarajan , A. Oszmiana , F. E. Rivera , H. K. Miller , J. E. Davenport , et al. 2011 NsaRS is a cell‐envelope‐stress‐sensing two‐component system of *Staphylococcus aureus* . Microbiology 157:2206–2219. 21565927 10.1099/mic.0.049692-0 PMC3167884

[mbo3286-bib-0036] Korsak, D. , S. Liebscher , and W. Vollmer . 2005 Susceptibility to antibiotics and beta‐lactamase induction in murein hydrolase mutants of *Escherichia coli* . Antimicrob. Agents Chemother. 49:1404–1409. 15793119 10.1128/AAC.49.4.1404-1409.2005 PMC1068617

[mbo3286-bib-0037] Kostakioti, M. , M. Hadjifrangiskou , and S. J. Hultgren . 2013 Bacterial biofilms: development, dispersal, and therapeutic strategies in the dawn of the postantibiotic era. Cold Spring Harb. Perspect. Med. 3:a010306. 23545571 10.1101/cshperspect.a010306 PMC3683961

[mbo3286-bib-0038] Kraft, A. R. , J. Prabhu , A. Ursinus , and J. V. Holtje . 1999 Interference with murein turnover has no effect on growth but reduces beta‐lactamase induction in *Escherichia coli* . J. Bacteriol. 181:7192–7198. 10572120 10.1128/jb.181.23.7192-7198.1999 PMC103679

[mbo3286-bib-0039] Kumari, H. , D. Balasubramanian , D. Zincke , and K. Mathee . 2014 Role of *Pseudomonas aeruginosa* AmpR on beta‐lactam and non‐beta‐lactam transient cross‐resistance upon pre‐exposure to subinhibitory concentrations of antibiotics. J. Med. Microbiol. 63:544–555. 24464693 10.1099/jmm.0.070185-0 PMC3973449

[mbo3286-bib-0040] Lamers, R. P. , J. F. Cavallari , and L. L. Burrows . 2013 The efflux inhibitor phenylalanine‐arginine beta‐naphthylamide (PAbetaN) permeabilizes the outer membrane of gram‐negative bacteria. PLoS One 8:e60666. 23544160 10.1371/journal.pone.0060666 PMC3609863

[mbo3286-bib-0041] Lee, M. , D. Hesek , L. I. Llarrull , E. Lastochkin , H. Pi , B. Boggess , et al. 2013 Reactions of all *Escherichia coli* lytic transglycosylases with bacterial cell wall. J. Am. Chem. Soc. 135:3311–3314. 23421439 10.1021/ja309036q PMC3645847

[mbo3286-bib-0042] Lee, M. , D. Hesek , B. Blazquez , E. Lastochkin , B. Boggess , J. F. Fisher , et al. 2015 Catalytic spectrum of the penicillin‐binding protein 4 of *Pseudomonas aeruginosa*, a nexus for the induction of beta‐lactam antibiotic resistance. J. Am. Chem. Soc. 137:190–200. 25495032 10.1021/ja5111706 PMC4304477

[mbo3286-bib-0043] Legaree, B. A. , and A. J. Clarke . 2008 Interaction of penicillin‐binding protein 2 with soluble lytic transglycosylase B1 in *Pseudomonas aeruginosa* . J. Bacteriol. 190:6922–6926. 18708507 10.1128/JB.00934-08 PMC2566182

[mbo3286-bib-0044] Li, X. Z. , L. Zhang , R. Srikumar , and K. Poole . 1998 Beta‐lactamase inhibitors are substrates for the multidrug efflux pumps of *Pseudomonas aeruginosa* . Antimicrob. Agents Chemother. 42:399–403. 9527793 10.1128/aac.42.2.399 PMC105421

[mbo3286-bib-0045] Lister, P. D. , D. J. Wolter , and N. D. Hanson . 2009 Antibacterial‐resistant *Pseudomonas aeruginosa*: clinical impact and complex regulation of chromosomally encoded resistance mechanisms. Clin. Microbiol. Rev. 22:582–610. 19822890 10.1128/CMR.00040-09 PMC2772362

[mbo3286-bib-0046] Liu, W. , N. Dong , and X. H. Zhang . 2012 Overexpression of *mltA* in *Edwardsiella tarda* reduces resistance to antibiotics and enhances lethality in zebra fish. J. Appl. Microbiol. 112:1075–1085. 22443589 10.1111/j.1365-2672.2012.05291.x

[mbo3286-bib-0047] Liu, Z. , H. Niu , S. Wu , and R. Huang . 2014 CsgD regulatory network in a bacterial trait‐altering biofilm formation. Emerg. Microbes Infect. 3:e1. 26038492 10.1038/emi.2014.1 PMC3913822

[mbo3286-bib-0048] Mark, B. L. , D. J. Vocadlo , and A. Oliver . 2011 Providing beta‐lactams a helping hand: targeting the AmpC beta‐lactamase induction pathway. Future Microbiol. 6:1415–1427. 22122439 10.2217/fmb.11.128

[mbo3286-bib-0049] Mizuno, T. 1979 A novel peptidoglycan‐associated lipoprotein found in the cell envelope of *Pseudomonas aeruginosa* and *Escherichia coli* . J. Biochem. 86:991–1000. 115860 10.1093/oxfordjournals.jbchem.a132631

[mbo3286-bib-0050] Mizuno, T. , and M. Kageyama . 1979 Isolation and characterization of a major outer membrane protein of *Pseudomonas aeruginosa*. Evidence for the occurrence of a lipoprotein. J. Biochem. 85:115–122. 104984 10.1093/oxfordjournals.jbchem.a132300

[mbo3286-bib-0051] Monteiro, C. , X. Fang , I. Ahmad , M. Gomelsky , and U. Romling . 2011 Regulation of biofilm components in *Salmonella enterica* serovar Typhimurium by lytic transglycosylases involved in cell wall turnover. J. Bacteriol. 193:6443–6451. 21965572 10.1128/JB.00425-11 PMC3232906

[mbo3286-bib-0052] Moya, B. , A. Dotsch , C. Juan , J. Blazquez , L. Zamorano , S. Haussler , et al. 2009 Beta‐lactam resistance response triggered by inactivation of a nonessential penicillin‐binding protein. PLoS Pathog. 5:e1000353. 19325877 10.1371/journal.ppat.1000353 PMC2654508

[mbo3286-bib-0053] Neidhardt, F. C. , J. Ingraham , and M. Schaechter . 1990 Physiology of the bacterial cell: a molecular approach. Sinauer Associates, Sunderland, MA.

[mbo3286-bib-0054] Ni, Y. , and R. R. Chen . 2004 Accelerating whole‐cell biocatalysis by reducing outer membrane permeability barrier. Biotechnol. Bioeng. 87:804–811. 15329939 10.1002/bit.20202

[mbo3286-bib-0055] Ni, Y. , and R. R. Chen . 2005 Lipoprotein mutation accelerates substrate permeability‐limited toluene dioxygenase‐catalyzed reaction. Biotechnol. Prog. 21:799–805. 15932259 10.1021/bp049575g

[mbo3286-bib-0056] Nikaido H., ed. 1996 Outer membrane. ASM Press, Washington, DC.

[mbo3286-bib-0057] Nikaido, H. 2003 Molecular basis of bacterial outer membrane permeability revisited. Microbiol. Mol. Biol. Rev. 67:593–656. 14665678 10.1128/MMBR.67.4.593-656.2003 PMC309051

[mbo3286-bib-0058] Nikaido, H. 2005 Restoring permeability barrier function to outer membrane. Chem. Biol. 12:507–509. 15911368 10.1016/j.chembiol.2005.05.001

[mbo3286-bib-0059] Payne, D. E. , N. R. Martin , K. R. Parzych , A. H. Rickard , A. Underwood , and B. R. Boles . 2013 Tannic acid inhibits *Staphylococcus aureus* surface colonization in an IsaA‐dependent manner. Infect. Immun. 81:496–504. 23208606 10.1128/IAI.00877-12 PMC3553799

[mbo3286-bib-0060] Plesiat, P. , J. R. Aires , C. Godard , and T. Kohler . 1997 Use of steroids to monitor alterations in the outer membrane of *Pseudomonas aeruginosa* . J. Bacteriol. 179:7004–7010. 9371446 10.1128/jb.179.22.7004-7010.1997 PMC179640

[mbo3286-bib-0061] Powell, A. J. , Z. J. Liu , R. A. Nicholas , and C. Davies . 2006 Crystal structures of the lytic transglycosylase MltA from *N. gonorrhoeae* and *E. coli*: insights into interdomain movements and substrate binding. J. Mol. Biol. 359:122–136. 16618494 10.1016/j.jmb.2006.03.023

[mbo3286-bib-0062] Priyadarshini, R. , D. L. Popham , and K. D. Young . 2006 Daughter cell separation by penicillin‐binding proteins and peptidoglycan amidases in *Escherichia coli* . J. Bacteriol. 188:5345–5355. 16855223 10.1128/JB.00476-06 PMC1540038

[mbo3286-bib-0063] Reid, C. W. , N. T. Blackburn , and A. J. Clarke . 2004 The effect of NAG‐thiazoline on morphology and surface hydrophobicity of *Escherichia coli* . FEMS Microbiol. Lett. 234:343–348. 15135542 10.1016/j.femsle.2004.03.047

[mbo3286-bib-0064] Rice, L. B. 2008 Federal funding for the study of antimicrobial resistance in nosocomial pathogens: no ESKAPE. J. Infect. Dis. 197:1079–1081. 18419525 10.1086/533452

[mbo3286-bib-0065] Ruiz, N. , B. Falcone , D. Kahne , and T. J. Silhavy . 2005 Chemical conditionality: a genetic strategy to probe organelle assembly. Cell 121:307–317. 15851036 10.1016/j.cell.2005.02.014

[mbo3286-bib-0066] Sailer, F. C. , B. M. Meberg , and K. D. Young . 2003 Beta‐lactam induction of colanic acid gene expression in *Escherichia coli* . FEMS Microbiol. Lett. 226:245–249. 14553918 10.1016/S0378-1097(03)00616-5

[mbo3286-bib-0067] Scheurwater, E. , C. W. Reid , and A. J. Clarke . 2008 Lytic transglycosylases: bacterial space‐making autolysins. Int. J. Biochem. Cell Biol. 40:586–591. 17468031 10.1016/j.biocel.2007.03.018

[mbo3286-bib-0068] Silhavy, T. J. , D. Kahne , and S. Walker . 2010 The bacterial cell envelope. Cold Spring Harb. Perspect. Biol. 2:a000414. 20452953 10.1101/cshperspect.a000414 PMC2857177

[mbo3286-bib-0069] van Straaten, K. E. , B. W. Dijkstra , W. Vollmer , and A. M. Thunnissen . 2005 Crystal structure of MltA from *Escherichia coli* reveals a unique lytic transglycosylase fold. J. Mol. Biol. 352:1068–1080. 16139297 10.1016/j.jmb.2005.07.067

[mbo3286-bib-0070] Sutterlin, H. A. , S. Zhang , and T. J. Silhavy . 2014 Accumulation of phosphatidic acid increases vancomycin resistance in *Escherichia coli* . J. Bacteriol. 196:3214–3220. 24957626 10.1128/JB.01876-14 PMC4135694

[mbo3286-bib-0071] Templin, M. F. , D. H. Edwards , and J. V. Holtje . 1992 A murein hydrolase is the specific target of bulgecin in *Escherichia coli* . J. Biol. Chem. 267:20039–20043. 1400320

[mbo3286-bib-0072] Thunnissen, A. M. , A. J. Dijkstra , K. H. Kalk , H. J. Rozeboom , H. Engel , W. Keck , et al. 1994 Doughnut‐shaped structure of a bacterial muramidase revealed by X‐ray crystallography. Nature 367:750–753. 8107871 10.1038/367750a0

[mbo3286-bib-0073] Tomasz, A. 1979 The mechanism of the irreversible antimicrobial effects of penicillins: how the beta‐lactam antibiotics kill and lyse bacteria. Annu. Rev. Microbiol. 33:113–137. 40528 10.1146/annurev.mi.33.100179.000553

[mbo3286-bib-0074] Tomasz, A. , A. Albino , and E. Zanati . 1970 Multiple antibiotic resistance in a bacterium with suppressed autolytic system. Nature 227:138–140. 4393335 10.1038/227138a0

[mbo3286-bib-0075] Uehara, T. , T. Dinh , and T. G. Bernhardt . 2009 LytM‐domain factors are required for daughter cell separation and rapid ampicillin‐induced lysis in *Escherichia coli* . J. Bacteriol. 191:5094–5107. 19525345 10.1128/JB.00505-09 PMC2725582

[mbo3286-bib-0076] Wang, Y. 2002 The function of OmpA in *Escherichia coli* . Biochem. Biophys. Res. Commun. 292:396–401. 11906175 10.1006/bbrc.2002.6657

[mbo3286-bib-0077] Wenderska, I. B. , M. Chong , J. McNulty , G. D. Wright , and L. L. Burrows . 2011 Palmitoyl‐DL‐carnitine is a multitarget inhibitor of *Pseudomonas aeruginosa* biofilm development. Chembiochem 12:2759–2766. 22045628 10.1002/cbic.201100500

[mbo3286-bib-0078] Wessel, A. K. , J. Liew , T. Kwon , E. M. Marcotte , and M. Whiteley . 2013 Role of *Pseudomonas aeruginosa* peptidoglycan‐associated outer membrane proteins in vesicle formation. J. Bacteriol. 195:213–219. 23123904 10.1128/JB.01253-12 PMC3553829

[mbo3286-bib-0079] World Health Organization . 2014 Antimicrobial resistance: global report on surveillance.

[mbo3286-bib-0080] Zamorano, L. , T. M. Reeve , L. Deng , C. Juan , B. Moya , G. Cabot , et al. 2010 NagZ inactivation prevents and reverts beta‐lactam resistance, driven by AmpD and PBP 4 mutations, in *Pseudomonas aeruginosa* . Antimicrob. Agents Chemother. 54:3557–3563. 20566764 10.1128/AAC.00385-10 PMC2934985

[mbo3286-bib-0081] Zamorano, L. , T. M. Reeve , C. Juan , B. Moya , G. Cabot , D. J. Vocadlo , et al. 2011 AmpG inactivation restores susceptibility of pan‐beta‐lactam‐resistant *Pseudomonas aeruginosa* clinical strains. Antimicrob. Agents Chemother. 55:1990–1996. 21357303 10.1128/AAC.01688-10 PMC3088256

[mbo3286-bib-0082] Zhang, Y. , Q. Bao , L. A. Gagnon , A. Huletsky , A. Oliver , S. Jin , et al. 2010 *ampG* gene of *Pseudomonas aeruginosa* and its role in beta‐lactamase expression. Antimicrob. Agents Chemother. 54:4772–4779. 20713660 10.1128/AAC.00009-10 PMC2976151

