# Supplementary material for: Loss of membrane‐bound lytic transglycosylases increases outer membrane permeability and β‐lactam sensitivity in Pseudomonas aeruginosa
Source: Microbiologyopen. 2015 Sep 15;4(6):879–95. doi: 10.1002/mbo3.286 (PMC4694138; doi:10.1002/mbo3.286)
Supplement: Supplementary file 1 — Table S1. Primer details for PCR confirmation of LT mutant strains. Table S2. MICs for all LT mutants of β‐lactam, fluoroquinolone, and glycopeptide antibiotics. Table S3. Summary of phenotypes associated with LT mutants. Figure S1. Loss of LTs does not cause growth defects. Bacterial growth (OD600 nm) was monitored every hour for 48 h using a plate‐based assay, described below. Curves are average of three biological replicates with three technical replicates each. Following the strain names in each legend are the growth rates per minute (min−1) and lag times (in min), respectively. Figure S2. Loss of LTs does not affect AmpC expression. Strains lacking the indicated LT were grown under basal (top panel) or AmpC‐inducing (bottom panel) conditions and immunoblotted using α‐AmpC antibodies, as described in the Materials and Methods section. Loss of LTs does not prevent either basal AmpC expression or AmpC induction. Figure S3. Magnesium supplementation does not affect bile salt sensitivity in mLT mutants. Bile salt assays were performed as described in the Materials and Methods section with and without MgCl2 (1 mmol/L final) supplementation. Bile salt sensitivity of the mltA/B/D/F/F2 and mltD/F/F2/slt mutants was unaffected by the addition of magnesium. Bars representing non‐MgCl2‐treated samples are the same as those used in Figure 3A. N = 3. Bars represent the mean ± SEM. [file MBO3-4-0879-s001.pdf]

Table S1. Primer details for PCR confirmation of LT mutant strains.

Gene[a]	Primer name[b]	Primer sequence (5'-3')
mltA	mltAFwd	TTT GAC GCA CCG TCG CGC TTT GCC CAA GC
	mltAmid	GCT GGA GGA ACG CAG GCT ATA GAC CTG CAT CC
	mltARev	GGC GTC ATT CAT CGT TTG AAT GGT TGC TGC C
mltB	mltBFwd	AGT ACA GCA TCA CCG GGT TAT CGA ACT CG
	mltBmid	GAC CAT CCT GTC GAT GAA GTG CTG TGC
	mltBRev	TTC GCC ATC GAG CAC TTG TTC ATC GAC TG
mltD	mltDFwd	CTT GAC CAC TCT TGA GGT CAT TTC TAG AAT CGG
	mltDmid	GCT CGA CCA CGT AGT GCA TGT AGA GG
	mltDRev	ATC AGG AAG GAG AGG CGA CGC ATC AGG
mltF	mltFFwd	TGC TTG CGG GCT GTA GCG AGG CGA AAG C
	mltFmid	CCT CGC GGG AAA GCT GGG CAT AGA GGT CG
	mltFRev	CGA TCT CGA TAT CCG TGG TTC CAG CGA GG
mltF2	mltF2Fwd	GCT TGT AGA ACA ATC CGT TAT AAT CGC AGA TCG
	mltF2mid	CGG CTG TTG AGG TAT TGC TCG AAG G
	mltF2Rev	GGA TAA GAT GGG CAC ACT ATA GGC AAG ATA ATT CG
slt	sltFwd	CGG TGA ACT TGA GCA GGG TCA TGG GC
	sltmid	AGA CGG CGT TGT TCG GTC AGG GAA GC
	sltRev	TCT TCA GCA GGG TTT TCT GGC CGT CG
sltG	sltGFwd	GCC ATG GAT GAC CGT TCA CCG TAC AAG G
	sltGmid	ACC GTG TAC TTG TCC ACG CCG TAG C
	sltGRev	TGC CAC AAC TTC GAG TTC ATG GAC TTC ACC
sltH	sltHFwd	CCG ATG GAA CCA CAT TGG CGA ACC
	sltHmid	CGA TCG AAG GTC TGC GCA TCG ATT CC
	sltHRev	ACC TGG AAT ACT TGT TTT GCC AGA TCC AGA CC

[a]Gene name refers to the gene that has been deleted.

[b]Fwd (forward) and Rev (reverse) PCR primers anneal to loci flanking the deleted gene and yield a truncated PCR product in knockout strains. "Mid" primers are specific for the deleted gene and yield no PCR product in knockout strains.

Table S2. MICs for all LT mutants of β-lactam, fluoroquinolone, and glycopeptide antibiotics

Strain	Minimum Inhibitory Concentration (μg/mL)[abc]						
	PP	CT	TZ	IP	CI	VA	PMB
PAO1	6 (4)	12 (8)	1 (2)	1	0.19	>256 (2,048)	(1)
ampC	2 (1)	4 (4)	— (0.5)	0.5	nd	nd	nd
dacB	64 (32)	>256 (128)	16 (16)	—	nd	nd	nd
dacB/slt	32 (8)	128 (64)	8 (8)	—	nd	nd	nd
slt	3 (1)	6 (4)	0.5 (1)	—	—	—	nd
slt (E503A)	4 (1)	6 (4)	0.5 (1)	—	nd	nd	nd
sltB1	16 (8)	24 (16)	— (—)	—	—	—	nd
sltB1/ampC	4 (2)	4 (4)	— (1)	0.5	nd	nd	nd
sltG	—	16	—	1.5	—	—	nd
sltH	12 (8)	32 (16)	— (—)	1.5	—	—	nd
sltH/ampC	4 (2)	16 (4)	— (1)	0.25	nd	nd	nd
slt/mltB	—	16	1.5	1.5	—	—	nd
slt/mltF	3 (1)	6 (4)	— (1)	—	—	—	nd
sltB1/mltB	32 (16)	96 (32)	1.5 (—)	0.75	—	—	nd
sltB1/slt	12 (—)	24 (—)	— (1)	0.5	—	—	nd
sltB1/G	16 (8)	24 (16)	1.5 (—)	—	—	—	nd
sltB1/H	32 (16)	48 (16)	1.5 (—)	—	—	—	nd
sltB1/G/mltB	24 (8)	32 (16)	— (—)	—	—	—	nd
sltB1/G/H	12 (8)	16 (—)	— (—)	—	—	—	nd
sltB1/G/H/mltB	12 (8)	16 (—)	1.5 (—)	—	0.125	— (—)	(—)
sltB1/G/H/slt	8 (—)	16 (—)	— (—)	0.75	—	— (—)	(—)
mltA	8	16	1.5	1.5	—	—	nd
mltB	12 (—)	32 (16)	1.5 (—)	1.5	—	—	nd
mltB (E162A)	8 (—)	16 (—)	— (—)	—	nd	nd	nd
mltB/ampC	8 (2)	16 (4)	— (—)	0.5	nd	nd	nd
mltD	12 (—)	24 (16)	— (—)	—	—	— (—)	(—)
mltD (E144A)	— (—)	16 (—)	— (—)	—	nd	nd	nd
mltD/ampC	— (2)	16 (4)	— (—)	0.5	nd	nd	nd
mltF	—	16	—	—	—	— (—)	(—)

mltF2	12 (—)	32 (16)	— (—)	—	—	—	nd
mltF2/ampC	— (—)	8 (4)	—	0.5	nd	nd	nd
mltB/F	8	—	—	—	—	—	nd
mltD/F	—	8	0.75	—	—	— (1,024)	(—)
mltB/D/F	4 (2)	6 (4)	0.75 (1)	—	—	—	nd
mltA/B/F	—	8	—	—	—	—	nd
mltB/F/F2	4 (2)	6 (4)	0.75 (1)	—	—	—	nd
mltD/F/F2	3 (1)	6 (4)	— (1)	0.75	—	— (512)	(—)
mltB/D/F/F2	3 (1)	4 (4)	— (1)	—	—	— (256)	(0.5)
mltD/F/F2/slt	1.5 (0.5)	2 (2)	0.5 (0.5)	0.5	0.125	128 (256)	(0.5)
mltA/B/D/F/F2	2 (0.5)	4 (4)	0.75 (0.5)	0.5	0.125	128 (128)	(0.5)

[a]Abbreviations: PP, piperacillin; CT, cefotaxime; TZ, ceftazidime; IP, imipenem; CI, ciprofloxacin; VA, vancomycin; PMB, Polymyxin B; nd, not done; —, MIC is same as wild type.

[b]MICs in blue are ≥2-fold higher than wild type, as confirmed by Etest and broth microdilution methods while those in red are ≥2-fold lower than wild type, as also confirmed by both methods.

[c]MIC values in parentheses were determined using broth microdilution while all other MIC values were determined by Etest.

Table S3. Summary of phenotypes associated with LT mutants.

Strain Name	LTs Present[a]	Phenotypes			
		β-lactam MICs	OM permeability	Osmotic stress sensitivity	Biofilm formation
PAO1	B1 G H Slt<br>A B D F F2				
slt	B1 G H<br>A B D F F2	↓			
slt (E503)	B1 G H Slt<br>A B D F F2	↓	nd	nd	nd
sltB1	G H Slt<br>A B D F F2	↑			
sltG	B1 H Slt<br>A B D F F2				
sltH	B1 G Slt<br>A B D F F2	↑			
slt/mltB	B1 G H<br>A D F F2				
slt/mltF	B1 G H<br>A B D F2	↓			
sltB1/mltB	G H Slt<br>A D F F2	↑			
sltB1/slt	G H<br>A B D F F2	↑			
sltB1/G	H Slt<br>A B D F F2	↑			
sltB1/H	G Slt<br>A B D F F2	↑			
sltB1/G/mltB	H Slt<br>A D F F2	↑			
sltB1/G/H	Slt<br>A B D F F2	↑			
sltB1/G/H/mltB	Slt<br>A D F F2	↑	↑		
sltB1/G/H/slt	A B D F F2				
mltA	B1 G H Slt<br>B D F F2		↑		

mltA (D350A)	B1 G H Slt<br>A B D F F2	nd		nd	nd
mltB	B1 G H Slt<br>A D F F2	↑			
mltB (E162A)	B1 G H Slt<br>A B D F F2		nd	nd	nd
mltD	B1 G H Slt<br>A B F F2	↑	↑		↓
mltD (E144A)	B1 G H Slt<br>A B D F F2			nd	nd
mltF	B1 G H Slt<br>A B D F2				
mltF2	B1 G H Slt<br>A B D F	↑			
mltB/F	B1 G H Slt<br>A D F2			↑	
mltD/F	B1 G H Slt<br>A B F2			↑	
mltB/D/F	B1 G H Slt<br>A F2	↓	↑	↑	
mltA/B/F	B1 G H Slt<br>D F2			↑	
mltB/F/F2	B1 G H Slt<br>A D	↓	↑	↑	
mltD/F/F2	B1 G H Slt<br>A B	↓	↑	↑	↑
mltB/D/F/F2	B1 G H Slt<br>A	↓	↑	↑	↑
mltD/F/F2/slt	B1 G H<br>A B	↓	↑	↑	↑
mltA/B/D/F/F2	B1 G H Slt	↓	↑	↑	↑

[a]Green squares represent sLTs; purple squares represent mLTs; yellow squares represent the active-site mutant version; nd, not done. Red arrows show a decrease compared to wild type, blue arrows, an increase compared to wild type.

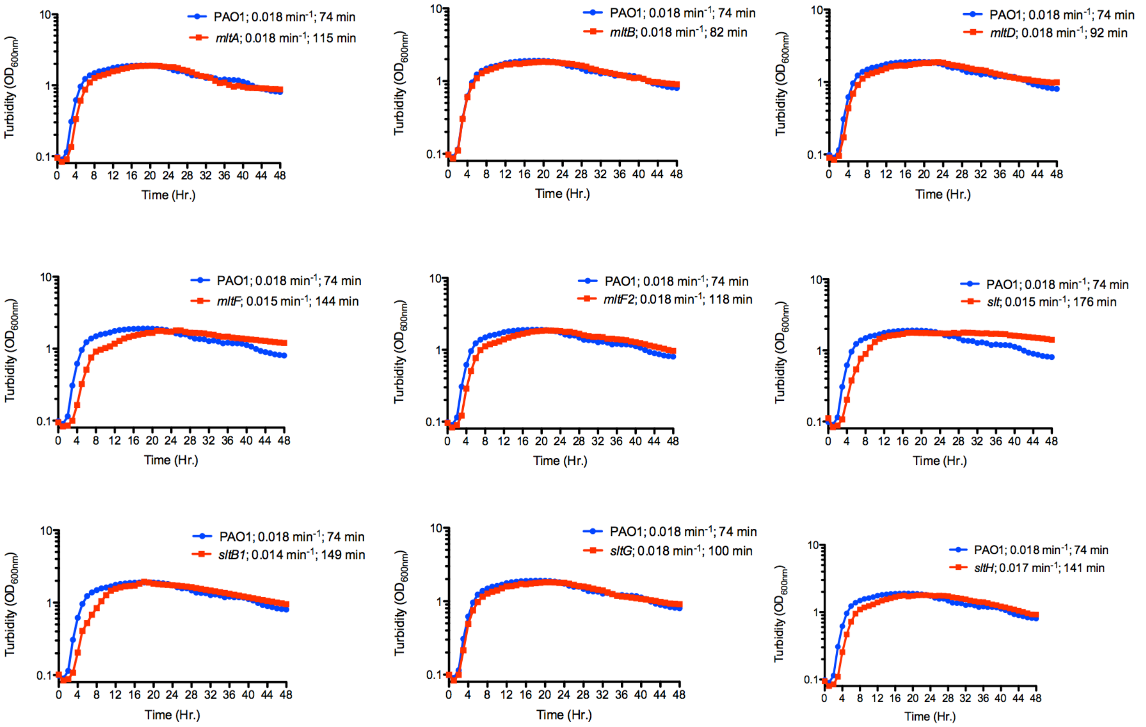

Figure S1. Loss of LTs does not cause growth defects. Bacterial growth (OD_{600nm}) was monitored every hour for 48 hours using a plate-based assay, described below. Curves are average of three biological replicates with three technical replicates each. Following the strain names in each legend are the growth rates per minute (min^{-1}) and lag times (in minutes), respectively.

Bacterial growth assays

Bacterial growth was monitored for 48 h using a plate-based assay. Overnight bacterial cultures (5mL LB) were subcultured 1:200 in fresh LB and 300 µL of each sample was added to triplicate wells of a sterile 100-well Honeycomb 2 plate (Oy Growth Curves Ab Ltd). Plates were incubated in a Bioscreen C plate reader (Oy Growth Curves Ab Ltd) for 48 h at 37°C with medium shaking. Turbidity readings were taken every hour at OD_{600nm}. Growth data were analyzed using GraphPad Prism software V5.0C. Growth rates and lag times were calculated using *GrowthRates* Software version 1.9 (Bellingham Research Institute), as previously described (Hall *et al.*, 2014. Growth rates made easy. Molecular Biology and Evolution **31:**232-238).

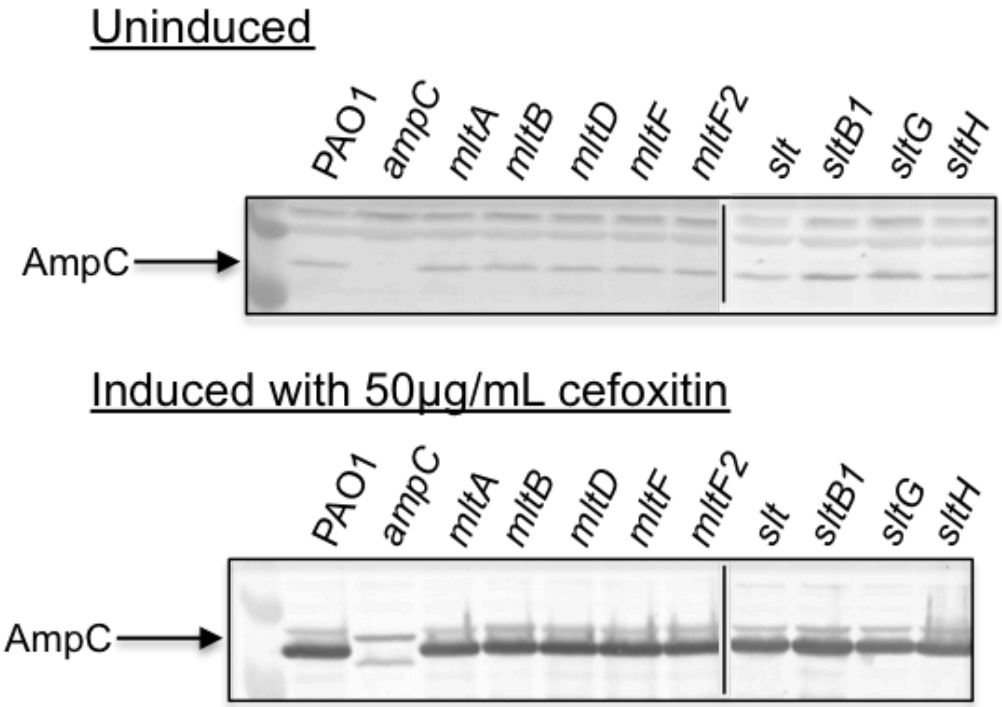

Figure S2. Loss of LTs does not affect AmpC expression. Strains lacking the indicated LT were grown under basal (top panel) or AmpC-inducing (bottom panel) conditions and immunoblotted using α-AmpC antibodies, as described in the Materials and Methods. Loss of LTs does not prevent either basal AmpC expression or AmpC induction.

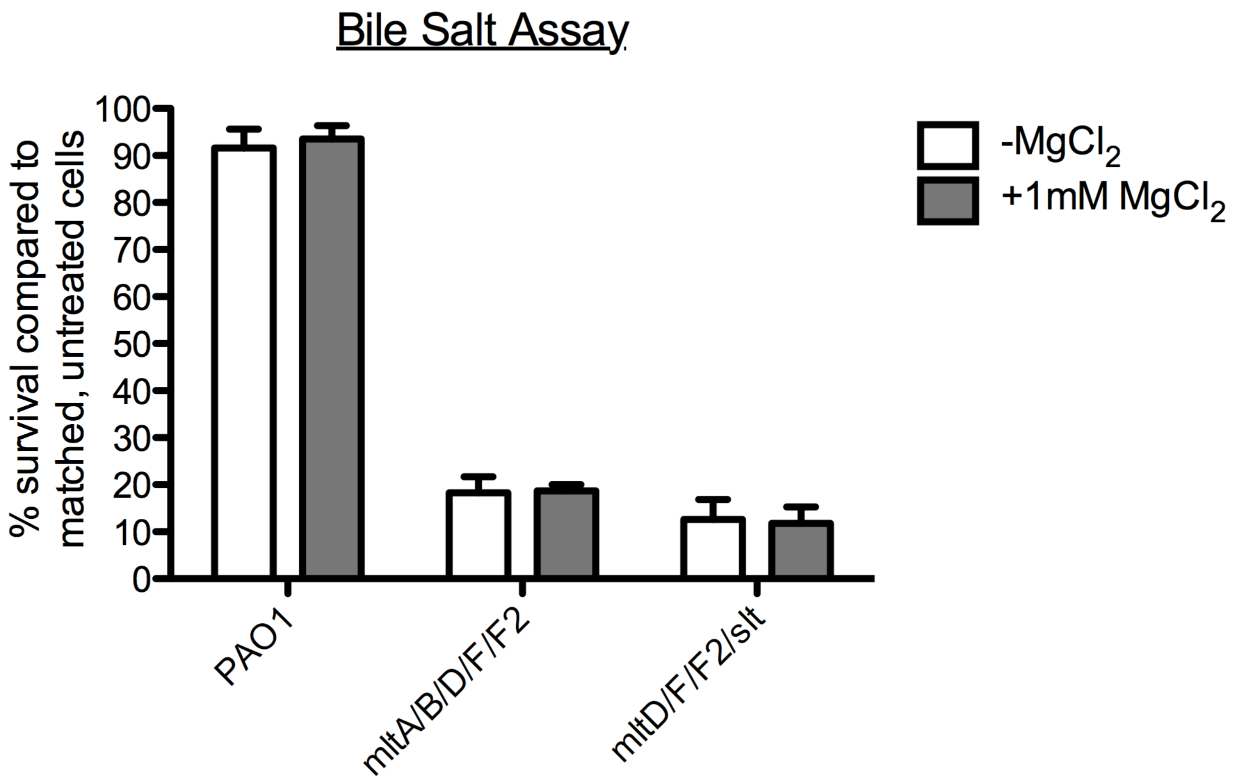

Figure S3. Magnesium supplementation does not affect bile salt sensitivity in mLT mutants. Bile salt assays were performed as described in the Materials and Methods with and without $MgCl_2$ (1mM final) supplementation. Bile salt sensitivity of the *mltA/B/D/F/F2* and *mltD/F/F2/slt* mutants was unaffected by the addition of magnesium. Bars representing non-$MgCl_2$-treated samples are the same as those used in **Figure 3a**. N=3. Bars represent the means ± SEM.
